# Stretchable Curvature Sensors for Motion Capture with Bending‐Stretching Coupling Deformation

**DOI:** 10.1002/advs.202514779

**Published:** 2025-12-16

**Authors:** Tairan Wang, Xinkai Xu, Shuang Li, Yuqun Lan, Kai Chen, Wei Li, Guangyuan Wang, Kuai Yu, Lijuan Sun, Yewang Su

**Affiliations:** ^1^ State Key Laboratory of Nonlinear Mechanics Institute of Mechanics Chinese Academy of Sciences Beijing 100190 P. R. China; ^2^ School of Engineering Science University of Chinese Academy of Sciences Beijing 100049 P. R. China; ^3^ Institute of Biomechanics and Medical Engineering AML Department of Engineering Mechanics Tsinghua University Beijing 100084 P. R. China; ^4^ China Academy of Space Technology Haidian District Beijing 100080 P. R. China; ^5^ State Key Laboratory of Intelligent Manufacturing Equipment and Technology Huazhong University of Science and Technology Wuhan 430074 P. R. China; ^6^ Zhongke Technology Achievement Transfer and Transformation Center of Henan Province Changyuan County Henan 453000 P. R. China

**Keywords:** decoupling, flexible electronics, human‐robot interaction, stretchable curvature sensor

## Abstract

Accurate curvature sensing is essential for robotic control, movement monitoring, and enhanced interactive experiences. Existing strategies rely either on stretchable strain sensors, which introduce non‐calibratable measurement errors due to unstable interfacial friction and slippage, or on non‐stretchable curvature sensors, which lack the stretchability required for wearable applications. Here, a stretchable curvature sensor is reported featuring a core component of a wave‐shaped symmetrical laminated structure. This structure achieves both stretchability and effective decoupling of bending from stretching, enabling accurate curvature measurement under frictional conditions. Slip‐simulation tests with smart gloves demonstrated a 93.6% reduction in measurement errors relative to conventional strain sensors. These results underscore the sensor's promise for applications in flexible wearable devices and soft robotics.

## Graphical abstract

Stretchable curvature sensors based on a wavy symmetric stacked‐layer structure (WSSLS) enable decoupled bending‐stretching detection with high linearity, repeatability, and durability. Integrated into smart gloves, the sensors achieved precise motion capture and human‐robot interaction, with slip‐simulation tests demonstrating a remarkable 93.6% reduction in measurement errors compared with conventional strain sensors. These results highlight their strong potential for next‐generation wearable electronics.

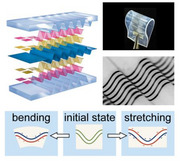

## 1 Introduction

Bending, as a fundamental deformation mode, is ubiquitous in both daily life and engineering applications.^[^ ^1^ ^]^ For example, humans bend joints to grasp and manipulate objects.^[^ ^2^, ^3^, ^4^, ^5^ ^]^ By replicating these bending behaviors, coupled with machine learning, humanoid robots can autonomously make decisions and handle hazardous objects in disaster rescue scenarios efficiently and safely, much like humans.^[^ ^6^ ^]^ In healthcare, prosthetic limbs support individuals with disabilities by bending mechanical knees to improve walking capability.^[^ ^7^, ^8^ ^]^ Meanwhile, by bending to varying extents, foldable screens enable mobile phones to switch between enhanced display performance and portability. Whether in robotic manipulation, medical devices or flexible electronics, accurate bending detection is essential for precise control, movement‐assistance monitoring and enhanced interactive experiences.^[^ ^9^, ^10^, ^11^, ^12^, ^13^, ^14^ ^]^

Curvature monitoring has been extensively researched over several decades, leading to thousands of publications.^[^ ^15^, ^16^ ^]^ Notwithstanding this vast body of work, only two strategies have been established to date. 1) Using stretchable strain sensors. This mechanism indirectly estimates bending deformation by using a stretchable strain sensor to measure surface strain.^[^ ^17^, ^18^, ^19^, ^20^ ^]^ This approach requires adhering the strain sensor directly to the surface of the skin, ensuring that the deformation of the sensor matches the skin's deformation exactly.^[^ ^21^ ^]^ However, because direct adhesion is often impractical, the strain sensor is typically attached to a fabric, such as a glove.^[^ ^22^ ^]^ Unstable interfacial friction and slippage at the skin‐fabric interface cause non‐calibratable measurement errors. 2) Using non‐stretchable curvature sensors. Non‐stretchable curvature sensors, including optical curvature sensors and those with planar laminated non‐stretchable structures,^[^ ^21^, ^23^ ^]^ possess high stiffness and lack stretchability. As a result, tensile deformation of the skin, fabrics, or other wearable materials induces sensor wrinkling and detachment, ultimately compromising the flexibility and comfort requirements of wearable applications. Furthermore, this inability to follow the stretching of soft materials or surfaces limits their application in various other fields such as wearable devices and smart sportswear.

In this study, we report a stretchable curvature sensor featuring a core component of an innovatively designed wave‐shaped symmetrical laminated structure (WSSLS), fabricated through a curved surface lithography technique. Distinct from previous structural strategies, such as single‐sided wavy designs that suffer from bending‐stretching coupling and planar laminated configurations that are intrinsically non‐stretchable, the WSSLS enables both stretchability and effective decoupling of bending from stretching, while also ensuring high measurement repeatability and linearity. More detailed direct benchmarking metrics are provided in Table S1 (Supporting Information). Furthermore, the sensor ensures consistent measurement under friction and slippage. In addition, the sensor offers enhanced softness, improved comfort, and effective accommodation of large‐gesture deformations. Benefiting from these advantages, the sensor precisely identifies human gestures and enables control of robotic arms and hexapod robots, advancing the development of human‐computer interaction.

## 2 Results and Discussion

### 2.1 Design, Fabrication, and Mechanism of the Stretchable Curvature Sensor

**Figure 1:**
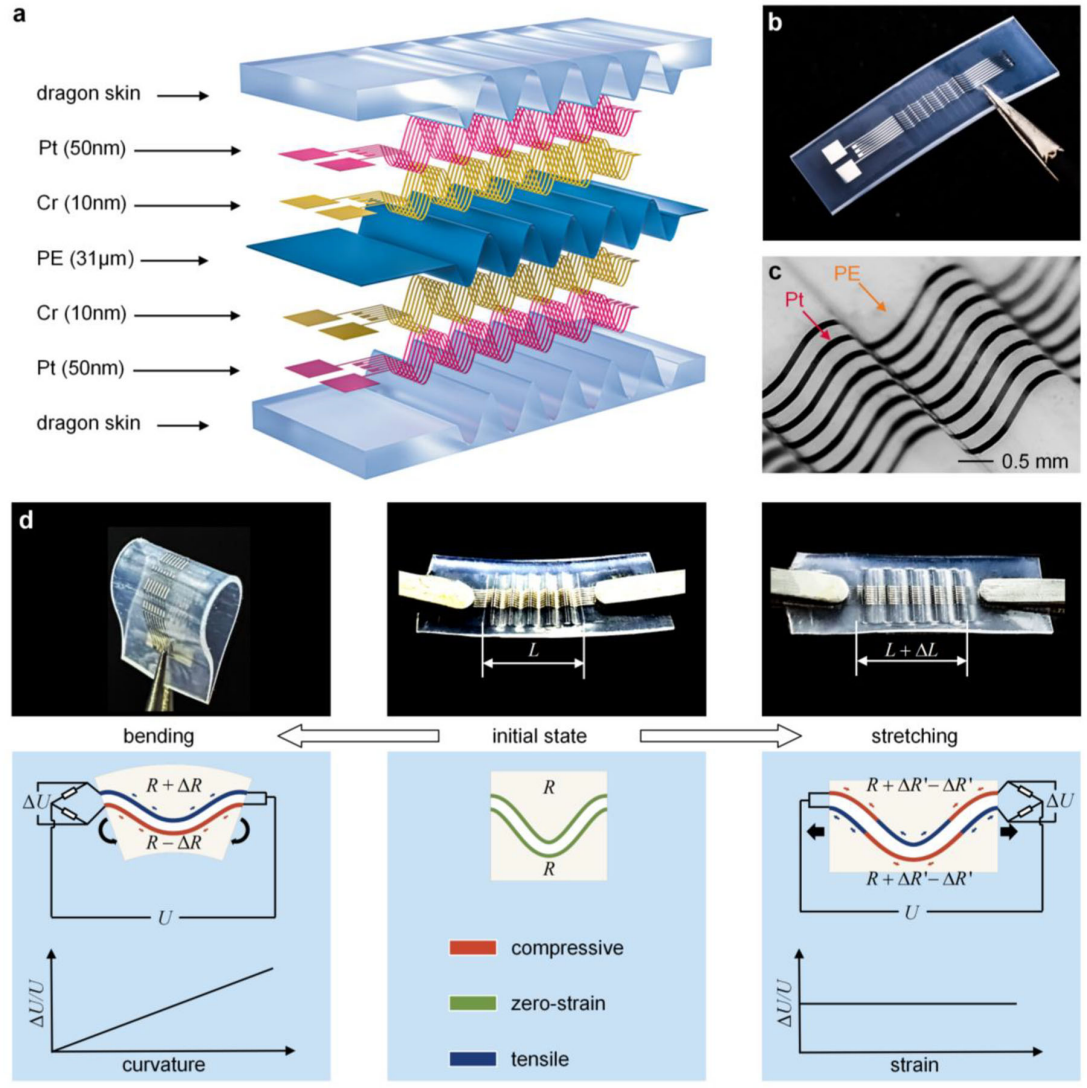
Design, visualization, and operating principle of the stretchable curvature sensor. a) Exploded view of the stretchable curvature sensor. b) Optical image of the fabricated curvature sensor. c) 3D digital microscope image of wave‐shaped metal conductive foils and the corresponding PE substrate. d) Schematic illustration of the sensor decoupling principle.

**Figure** **1**a illustrates the multilayer configuration of the stretchable curvature sensor, which consists of a WSSLS and encapsulation layers (Dragon skin 00–30, Smooth‐On, USA). The WSSLS consists of two sets of wave‐shaped metal conductive foils (platinum and chrome), a wave‐shaped polyethylene (PE) substrate, and four external leads connected via conductive epoxy resin adhesive. In this study, platinum (Pt) was selected as the sensing material owing to its excellent linearity, signal stability, environmental robustness, and fabrication compatibility. Although metallic materials possess a low elastic limit (≈0.5%), which constrains stretchability, they provide superior reliability in practical applications requiring high linearity, repeatability, and durability. Alternatively, non‐metallic composite materials, such as MXene‐based nanocomposites with molecular‐level crack modulation and reversible bonding networks, offer ultrahigh sensitivity and large stretchability but generally suffer from poor linearity and limited environmental stability, making them unsuitable for long‐term monitoring. Therefore, this work focuses on practical applications, and Pt was selected as the sensing material. The curvature sensor is demonstrated in Figure 1b, while Figure 1c presents a 3D digital microscope image (4KHB, AMEC, China) of the wave‐shaped metal conductive foils and the wave‐shaped PE substrate.

A feasible approach for fabricating WSSLS is to shape planar conductive foils into wavy configurations. Nevertheless, this process leads to excessive deformation of the conductive foils resulting in rupture.^[^ ^24^ ^]^ To effectively overcome this challenge, we have proposed an innovative curved surface lithography technique that combines the lift‐off and thermoplastic techniques, with details provided in Figure S1 (Supporting Information). The thermoplastic process was introduced between the development and magnetron sputtering steps, transforming the planar substrate into an out‐of‐plane wavy substrate. Metal was deposited onto the wavy substrate using magnetron sputtering, and the photoresist was subsequently stripped. Since the conductive foils are fabricated by magnetron sputtering on the wavy substrate, the resulting wavy conductive foils are free from initial stress, thereby preventing rupture of the sensor during the preparation process and substantially enhancing its stability and reliability.

The dual‐layer strain decoupling (DSD) mechanism takes advantage of the different responses of the two conductive foils, enabling the decoupling of bending from stretching signals in the stretchable curvature sensor (Figure 1d). Bending‐stretching decoupling refers to ensuring that bending‐induced signals are not contaminated by tensile strain, which is a common issue in single‐sided wavy or stretchable strain sensors. In the initial state, both the top and bottom conductive foils are strain‐free, with identical resistance denoted as *R*. When the sensor undergoes bending, the top conductive foil is subjected to tensile strain, while the bottom conductive foil experiences compressive strain. The resistance variations of the two conductive foils are identical, each denoted as Δ*R*, as illustrated in the left subfigure. The resistances of the two foils change to (*R* + Δ*R*) and (*R* − Δ*R*), respectively. The resistance changes caused by bending are converted into output voltage variations through connection to a Wheatstone bridge. The resistance change of the conductive foil exhibits a linear relationship with the relative variation in output voltage, as expressed by:

(1) $$\frac{\Delta U}{U}=\frac{\Delta R}{2R}$$

where *U* is the input voltage and Δ*U* is the output voltage variation. The derivation is detailed in Note S1 and illustrated in Figure S2 (Supporting Information). When the sensor is stretched, the substrate undergoes pure bending, resulting in centrosymmetric deformation over a half‐period, as shown in the right subfigure of Figure 1d. Under such deformation, part of the conductive foil is subjected to stretching, while the other part is subjected to compression. Due to the centrosymmetric nature of the deformation, the conductive foil experiences equal resistance variation in its stretched and compressed regions, each denoted as $\Delta R^{\prime }$. The resistance changes are $-\Delta R^{\prime }$ in the red region and $+\Delta R^{\prime }$ in the blue region. The total resistance after deformation remains $R+\Delta R^{\prime }-\Delta R^{\prime }=R$, consistent with its initial value. This ensures the sensor's output voltage remains unchanged, thereby maintaining accurate bending measurements even under simultaneous stretching and bending.

### 2.2 Mechanical and Electrical Characterizations of the WSSLS

**Figure 2:**
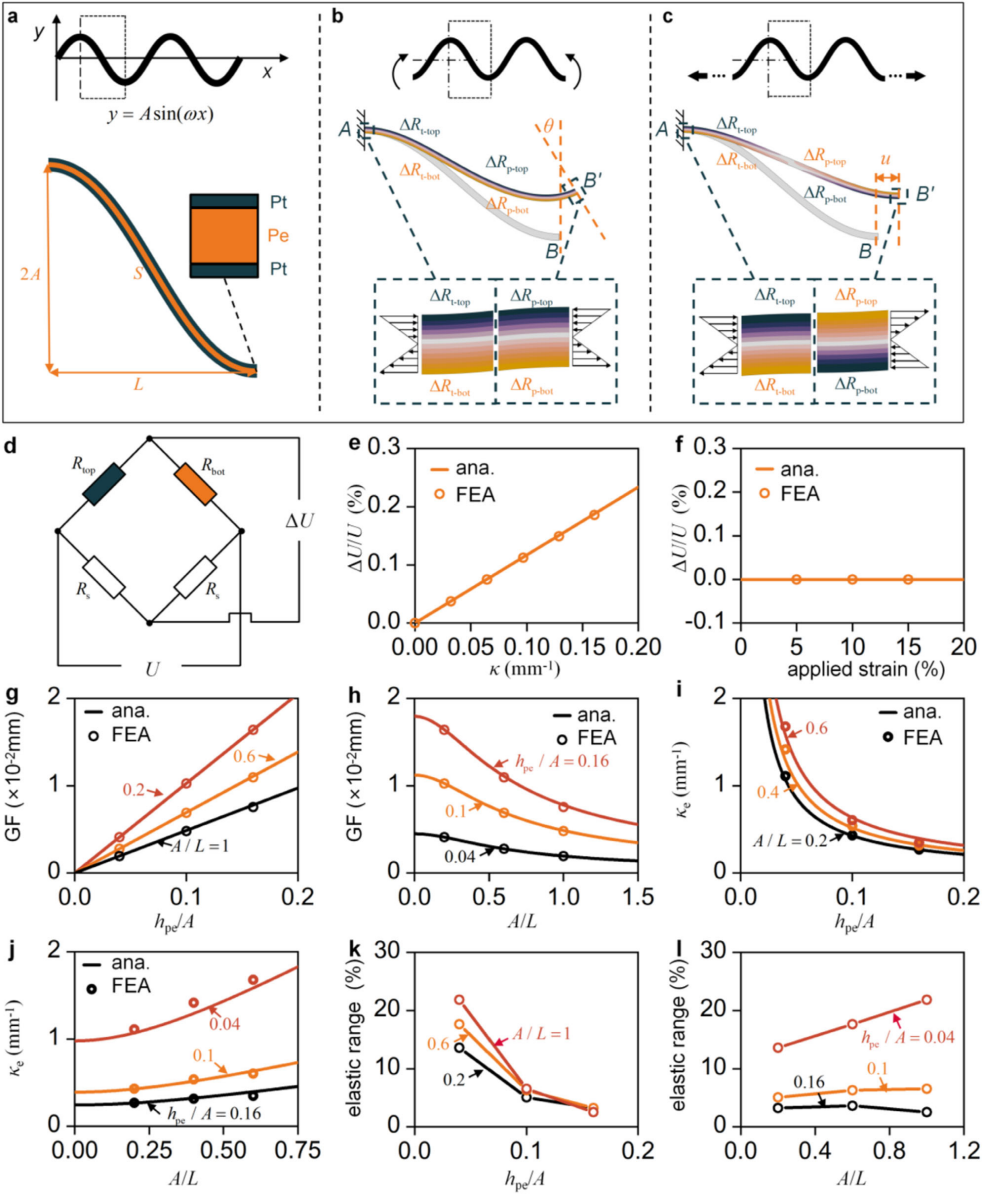
Theoretical modeling and FEA of the wave‐shaped symmetrical laminated structure (WSSLS). a) Geometric parameters of the WSSLS. b) Deformation of the WSSLS under bending. c) Deformation of the WSSLS under stretching. d) Schematic of the Wheatstone bridge circuit. e) Relative voltage change of the WSSLS as a function of applied curvature. f) Relative voltage change of the WSSLS as a function of applied strain. (g) GF as a function of *h*_pe_/*A* with *A*/*L* = 0.2, 0.6, and 1. h) GF as a function of *A*/*L* with *h*_pe_/*A* = 0.04, 0.1, and 0.16. i) κ_e_ as a function of *h*_pe_/*A* with *A*/*L* = 0.2, 0.4, and 0.6. j) κ_e_ as a function of *A*/*L* with *h*_pe_/*A* = 0.04, 0.1, and 0.16. k) Elastic range as a function of *h*_pe_/*A* with *A*/*L* = 0.2, 0.6, and 1. l) Elastic range as a function of *A*/*L* with *h*_pe_/*A* = 0.04, 0.1, and 0.16.

A mechanical model of the WSSLS was established to quantitatively analyze the influence of design parameters and to refine the design strategy. **Figure** **2**a illustrates the WSSLS with a sinusoidal configuration. Upon establishing a coordinate system, the shape function can be expressed as *y* = *A*sin(ω*x*). An enlarged view of half a period of the WSSLS is defined as the unit cell. The thickness of the substrate is denoted as *h*_pe_, and that of the conductive foil as *h*_pt_. The total thickness, given by *h* = *h*_pe_ + 2*h*_pt_ ≈ *h*_pe_, is treated as a constant in the calculations, and its variation is considered negligible. In the unit cell, *A* denotes the amplitude, *L* denotes half of the period length, and the arc length of the semi‐period is given by S=∫L/L223L/3L221+(y′)2dx. Figure 2b,c show the deformation of the WSSLS under bending and stretching loads, respectively. As shown in Figure 2b, a single period of the WSSLS is analyzed by leveraging its geometric symmetry. With the left end A fixed, the segment AB deforms into AB’ under an applied bending moment at the bottom end B. *R*_top_ and *R*_bot_ represent the resistances of the top and bottom conductive foils, while *R*_t_ and *R*_p_ correspond to the resistances of the trough and peak conductive foils. Under the applied bending load, the post‐deformation resistances are expressed as follows:

(2) $$\begin{array}{ccc} R_{\mathrm{top}} & = & \left(R_{t-\mathrm{top}}-\Delta R\right)+\left(R_{p-\mathrm{top}}-\Delta R\right),\\ R_{\mathrm{bot}} & = & \left(R_{t-\mathrm{bot}}-\Delta R\right)+\left(R_{p-\mathrm{bot}}-\Delta R\right) \end{array}$$

The configuration of the Wheatstone bridge is illustrated in Figure 2d, where *R*_s_ is a constant resistor. Under the aforementioned conditions, the output voltage of the Wheatstone bridge varies accordingly. The relationship between the sensor curvature and resistance is derived as follows:

(3) $$\kappa =\frac{\Delta R}{R}\frac{2S}{\left(1+2\mu \right)Lh}$$

wherei μ represents the Poisson's ratio of the conductive foil. The derivation is detailed in Note S2 (Supporting Information) and illustrated in Figure S3 (Supporting Information). By combining Equations 1 and 3, the relationship between the sensor curvature κ and Δ*U*/*U* is derived as follows:

(4) $$\frac{\Delta U}{U}=\frac{\left(1+2\mu \right)Lh}{4S}\kappa$$

The gauge factor (GF) of the curvature sensor, defined as the derivative of Δ*U*/*U* with respect to κ, is expressed as^[^ ^25^, ^26^, ^27^, ^28^ ^]^:

(5) $$\mathrm{GF}=\frac{d}{d\kappa }\left(\frac{\Delta U}{U}\right)=\frac{\left(1+2\mu \right)Lh}{4S}$$

The analytical relationship between the GF and strain (ε) is derived based on Equation (5), and the full derivation is provided in Note S3 (Supporting Information). The curvature of the WSSLS is defined as κ_e_ at the onset of plastic deformation in the conductive foil, and the corresponding elongation is referred to as elastic range. ε_Pt,max_ denotes the elastic limit strain of platinum, which is assumed to be 0.5% in this study. The expression for κ_e_ is given as:

(6) $$\kappa_{e}=\frac{2S\varepsilon_{\mathrm{Pt},\max}}{Lh}$$

The derivation is detailed in Note S4 (Supporting Information). As shown in Figure 2c, under the applied stretching load, the post‐deformation resistances are given by:

(7) $$\begin{array}{ccc} R_{\mathrm{top}} & = & \left(R_{t-\mathrm{top}}-\Delta R\right)+\left(R_{p-\mathrm{top}}+\Delta R\right)=constant,\\ R_{\mathrm{bot}} & = & \left(R_{t-\mathrm{bot}}+\Delta R\right)+\left(R_{p-\mathrm{bot}}-\Delta R\right)=constant \end{array}$$

Both the initial resistances of the top and bottom conductive foils and the resistance changes (Δ*R*) resulting from deformation are identical. As a result, the resistance values of *R*_top_ and *R*_bot_ remain constant before and after deformation, leading to no change in the output voltage Δ*U*/*U*.

The relationship between the output voltage Δ*U*/*U* and the curvature κ under bending is obtained from Equation 3, indicating that Δ*U*/*U* is proportional to κ, as shown in Figure 2e. Under stretching, Equation 7 indicates that Δ*U*/*U* remains constant throughout the deformation process, as shown in Figure 2f. Subsequently, the bending and stretching behaviors of the WSSLS were simulated using finite element analysis (FEA), and the corresponding electrical responses were obtained. The strong agreement between the FEA‐predicted responses and the theoretical results confirms the reliability of the theoretical model as a foundation for optimization‐oriented design. Furthermore, the results reveal a highly linear relationship between the electrical response and curvature.

To further optimize the sensor's performance, we investigated the influence of substrate thickness and geometric dimensions on the GF, κ_e_, and elastic range. The dimensionless parameter *h*_pe_/*A* was defined to characterize the relative substrate thickness, while *A*/*L* was introduced to represent the aspect ratio of the wave structure. The expressions for GF and κ_e_ are provided in Equations 5 and 6, respectively. GF increases monotonically with increasing *h*_pe_/*A*, and decreases monotonically with increasing *A*/*L*, as depicted in Figure 2g, h. *K_e_* decreases monotonically with increasing*h*_pe_/*A* and increases monotonically with increasing *A*/*L*, as shown in Figure 2i,j. The simulation results of GF and κ_e_ obtained through FEA show good agreement with the theoretical predictions. This consistency confirms the reliability of the theoretical analysis, enabling the sensor design to be tailored to specific application scenarios. Subsequently, the effects of the two parameters on the elastic range were examined through FEA, and the results are shown in Figure 2k,l. The results reveal a clear trend: the elastic range decreases monotonically with increasing *h*_pe_/*A*, while it increases monotonically with increasing *A*/*L*. As the *h*_pe_/*A* value increases, the substrate becomes thicker. Under identical deformation conditions, this results in greater surface strain, leading to a larger resistance change and an increased GF. However, the elevated surface strain also makes the conductive foil more susceptible to plastic deformation, thereby reducing both the κ_e_ and the elastic range. On the other hand, as the value of *A*/*L* increases, the wavy structure becomes narrower. Under identical surface strain, this results in a higher corresponding curvature, which leads to a decrease in the GF and an increase in the κ_e_. Meanwhile, enhanced structural compactness contributes to an increased elastic range.

### 2.3 Mechanical and Electrical Characterization of Stretchable Curvature Sensors with Encapsulation Layers

**Figure 3:**
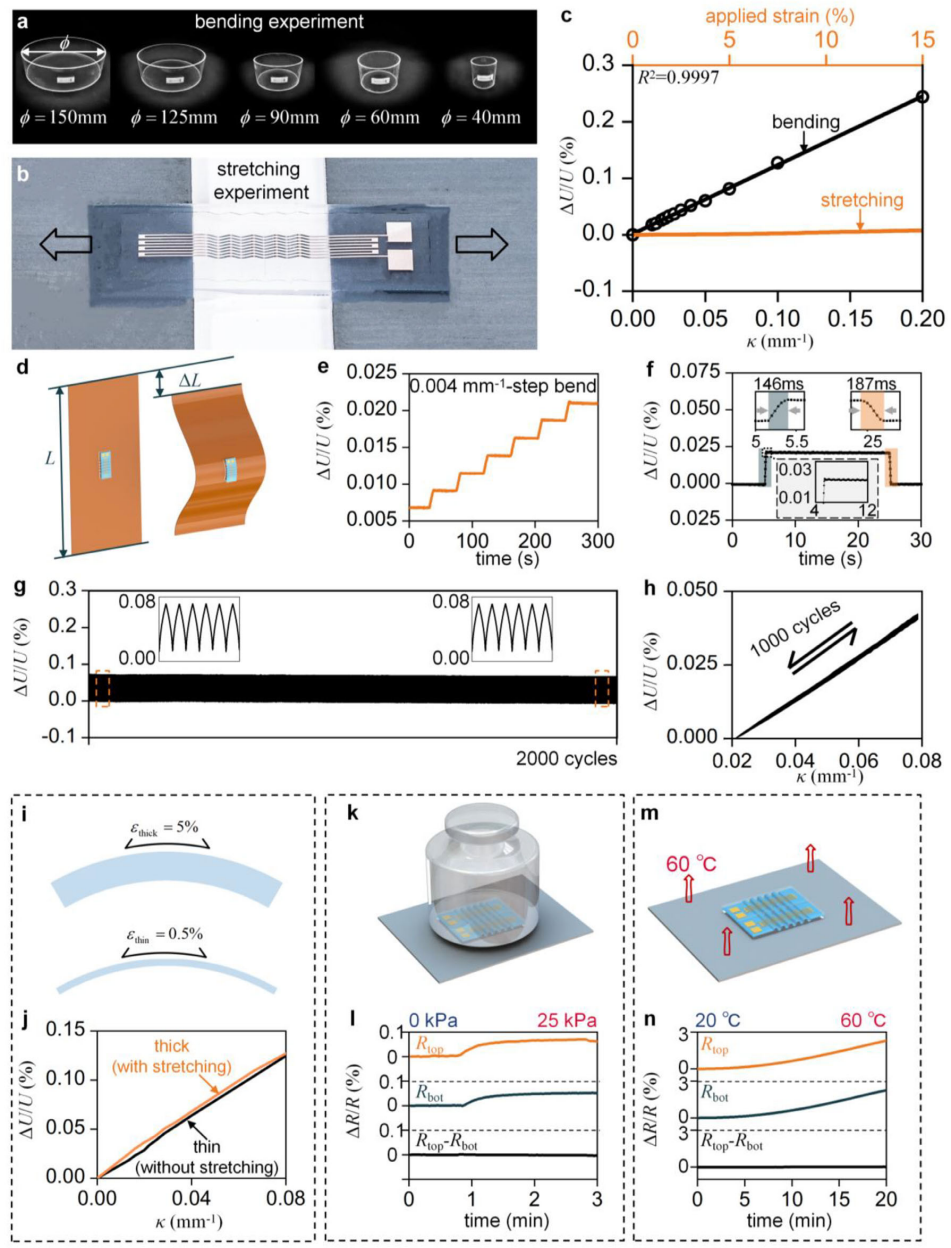
Electrical characterization of curvature sensors based on the WSSLS. a) Bending experiment of the curvature sensor. b) Stretching experiment of the curvature sensor. c) Relative voltage change of curvature sensors as a function of applied curvature and strain. d) Principle of dynamic performance testing. e) Relative voltage change under incrementally applied curvature with a step size of 0.004 mm^−1^. f) Rapid response of the curvature sensor to applied curvature (0.01 mm^−1^). Insets: Magnified views of response and recovery regions. g) Electrical stability of the curvature sensor under 2000 cycles at a curvature of 0.06 mm^−1^. h) Relative voltage change as a function of applied curvature under cyclic loading (1000 cycles). i) Schematic diagram of the bending‐stretching coupling test. j) Relative voltage change under combined stretching and bending. k) Validation of pressure self‐compensation capability. l) Resistance changes under applied pressure. m) Validation of temperature self‐compensation capability. n) Resistance changes under temperature variation.

The complete stretchable curvature sensor consists of the WSSLS and encapsulation layers, as illustrated in Figure 1a,b. The mechanical and electrical characterizations of the WSSLS are well preserved due to the high elasticity and low elastic modulus of the encapsulation materials. Figure 3a illustrates the bending experiment, in which the sensor is mounted on acrylic cylinders with diameters ranging from 10  to 150 mm. The discrete relationship between the electrical response and the applied curvature is subsequently obtained. Figure 3b illustrates the stretching experiment, in which both ends of the sensor are fixed to a tensile testing machine, while the central segment remains freestanding. The relationship between the electrical response and the applied strain is subsequently determined. To facilitate the stretching test, the sensor was designed with six periods, featuring an overall width of 6.8  and a length of 25 mm. Figure 3c presents the sensor's electrical responses under both stretching and bending, with the orange line representing the stretching data and black hollow circles indicating the bending data. A fitted curve for the bending data is shown as a solid black line. The relationship between Δ*U*/*U* and curvature exhibits high linearity (*R* ^2^ = 0.9997), outperforming previously reported curvature sensors.^[^ ^29^ ^]^ Moreover, the signal induced by stretching is negligible compared to that generated by bending.

To evaluate the dynamic performance of the curvature sensor, an experimental protocol based on the buckling behavior of polyimide (PI) films was developed for the quantitative and continuous assessment of the sensor's dynamic response (Figure 3d). The sensor was centrally mounted on a PI film (15 cm×10 cm × 100 µm), with both ends of the film clamped to a mechanical stretching apparatus. Compressive loading was then applied to induce controlled Euler buckling. During this process, the curvature at the film's center was accurately calculated based on the end displacement and film length, enabling quantitative and continuous curvature measurement. According to Euler buckling theory, the radius of curvature at the center of the buckled film is given by^[^ ^30^, ^31^ ^]^:

(8) $$\frac{1}{\kappa }=\frac{L}{2\pi }\sqrt{\frac{L}{\Delta L}}$$

In this equation, *L* denotes the original length of the undeformed PI film, and Δ*L* denotes the displacement at the upper end of the film. The relationship between the sensor's curvature and its electrical response can be derived analytically. Owing to its symmetrical structure, the sensor reliably detects both positive and negative curvatures, as depicted in Figure S4 (Supporting Information). Figure 3e illustrates the electrical response under an extremely small curvature (< 0.5%κ_e_). The curvature sensor was subjected to curvatures of 0.004, 0.008, 0.012, 0.016, 0.020, and 0.024 mm^−1^. The corresponding electrical response exhibits distinct step‐like features, indicating high resolution and enabling accurate detection of subtle deformations. The dynamic performance of the sensor is shown in Figure 3f. When a curvature of 0.01 mm^−1^ is applied, the electrical response increases sharply, reaching a response time of ≈146 ms. After being maintained for 20 s and subsequently released, the electrical response promptly recovers within ≈187 ms. The actual response and recovery times are calculated by subtracting the curvature transition time from the measured duration. The transition time is ≈46 ms. As shown in the subfigure, negligible hysteresis and overshoot are observed, indicating improved performance compared to most previously reported curvature sensors.^[^ ^29^ ^]^ When curvature was applied at four different loading speeds (0.5, 1, 2, and 4 mm s^−1^), the sensor exhibited highly consistent electrical responses, demonstrating excellent response uniformity across varying loading rates (Figure S5; Supporting Information). This remarkable dynamic stability is critical for practical applications. Repeatability was evaluated by subjecting the sensor to 2000 consecutive loading‐unloading cycles under a curvature of 0.06 mm^−1^, as shown in Figure 3g. The results under higher cycle numbers are provided in Figure S6 (Supporting Information). The electrical response at the end of the test shows no significant drift compared to the initial response. In our designed wave‐shaped symmetrical laminated structure, the conductive foils on both sides of the substrate undergo identical strain and uniform deformation, keeping the conductive foils well within the elastic limit. The relationship between curvature and electrical response over 1000 loading cycles (Figure 3h) demonstrates good consistency and strong linearity. The consistent electrical response under applied curvatures of 0.034, 0.028, and 0.019 mm^−1^ (Figure S7 (Supporting Information)) further demonstrates the high repeatability of the fabricated sensor. This stability is attributed to the fact that each material point of the WSSLS deforms along a certain path and experiences neither unstable contact resistance nor plastic deformation during repeated loading‐unloading cycles, thereby ensuring the repeatability of the electrical response. Such high repeatability is essential for long‐term dynamic applications and repetitive operational environments requiring repeated measurements.

According to the mechanics of materials, the surface strain of a 1 mm‐thick film at a curvature of 0.1 mm^−1^ is ≈5%, as shown in Figure 3i. The deformation of the curvature sensor attached to the thick film involves a complex coupling of stretching and bending. As shown in Figure 3j, the electrical responses of curvature sensors attached to both thick and thin substrates are nearly identical. This result indicates that the curvature sensor can maintain high‐precision curvature measurement even under stretching conditions, a capability that conventional strain sensors inherently lack due to their inability to decouple bending and stretching.^[^ ^22^ ^]^ This characteristic is particularly important for practical applications involving coupled stretching and bending deformations, such as in soft robotics. Eliminating the influence of environmental temperature and pressure is equally critical for the practical deployment of stretchable curvature sensors. The symmetrical structure of the WSSLS imparts the sensor with self‐compensation capabilities for both pressure and temperature variations. Both pressure and temperature variations induce identical resistance changes in the two conductive foils. These effects are eliminated by the Wheatstone bridge, without impairing the sensor's curvature detection capability. To validate the pressure self‐compensation capability, a 1 kg weight was applied to the curvature sensor during testing, as shown in Figure 3k. Under an applied pressure of 25 kPa, the resistance of the foils changes by ≈ 0.05%, whereas the resistance difference remains nearly unchanged (Figure 3l). To evaluate the temperature self‐compensation capability, the sensor was gradually heated to 60 °C in a temperature‐controlled chamber (Figure 3m). When the temperature increased by 40 °C, the resistance of the foils changed by ≈2.2%, whereas the resistance difference remained nearly unchanged (Figure 3n). To demonstrate the structural stability from a practical perspective, we conducted long‐term durability testing and stability evaluations under varying environmental conditions, including liquid immersion, low‐temperature (4 °C), and high‐temperature (50 °C) tests. These experiments showed negligible deviation in curvature response, confirming excellent durability and stability under realistic wearable conditions. The details are provided in Note S5 (Supporting Information) and illustrated in Figure S8 (Supporting Information).

### 2.4 Comparison with Conventional Sensing Strategies and Applications to Dynamic Curvature Monitoring

**Figure 4:**
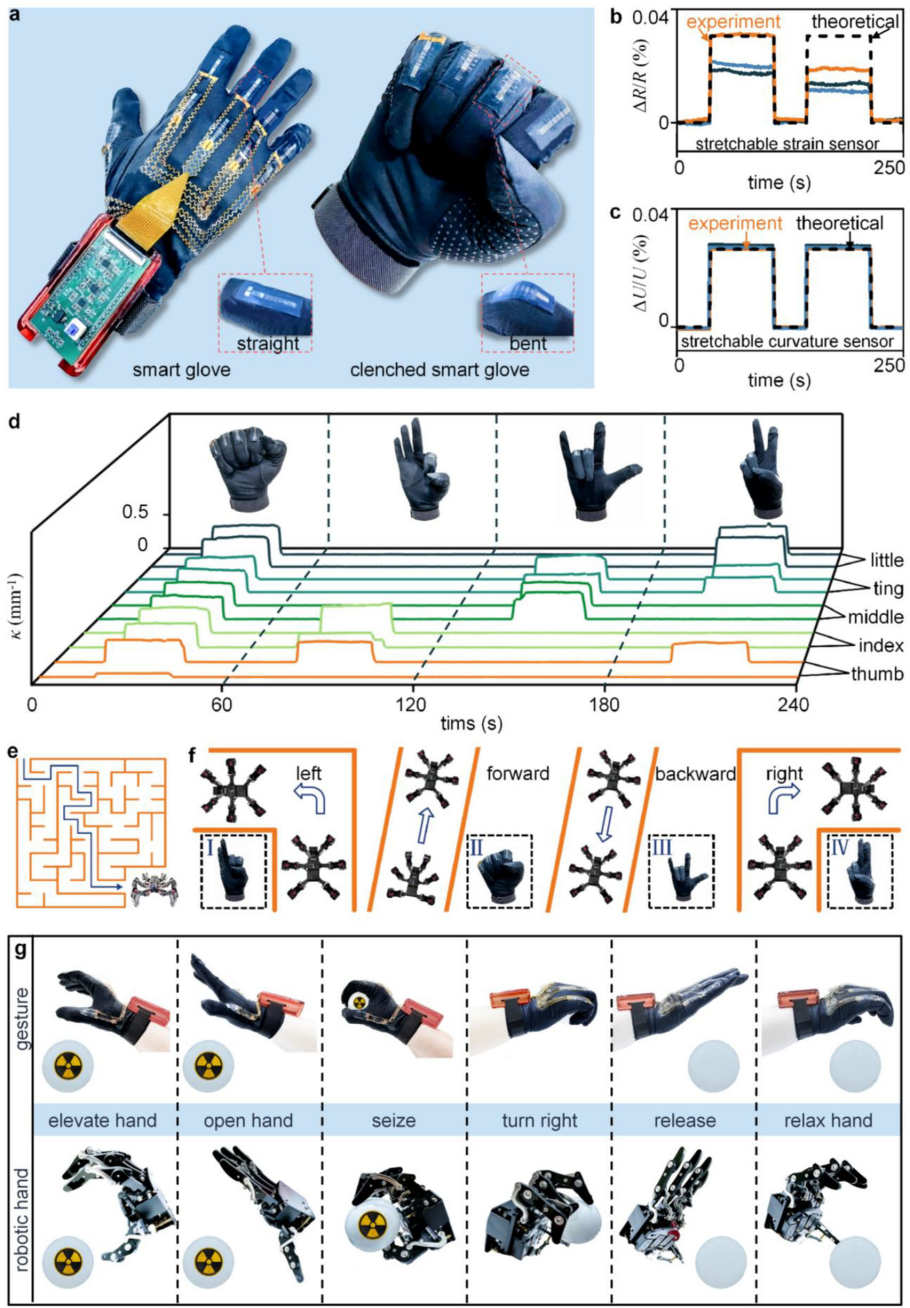
Gesture recognition and robotic control enabled by stretchable curvature sensors. a) Sensor configuration on the glove. b) Theoretical and experimental results of stretchable strain sensors under interfacial friction and slippage. c) Theoretical and experimental results of stretchable curvature sensors under interfacial friction and slippage. d) Four distinct gestures and corresponding real‐time curvature data. e) Gesture‐based control of a hexapod robot navigating a maze. f) Gestures corresponding to four commands: turning left, forward, backward, and turning right. g) Gesture‐based control of a mechanical hand for grasping hazardous objects.

To illustrate the potential application of stretchable curvature sensors in wearable devices, we developed a smart glove capable of accurately detecting finger curvature for gesture recognition (Figure 4a). Integrated into a flexible glove with precise alignment to the finger joints, each sensor is connected via a serpentine flexible printed circuit board to a data module positioned at the wrist. This module processes the resistance signals and wirelessly transmits control commands to robotic systems (Figures S9 and S10, Supporting Information).

To evaluate the performance advantage of the developed sensor, comparative experiments were conducted against the two conventional strategies. In comparison with the conventional stretchable strain sensor strategy,^[^ ^20^ ^]^ we performed slip‐simulation tests using soft silicone (Ecoflex 00–30, Smooth‐On, USA) and fabric, simulating the uncontrolled interfacial friction and slippage that occur at the fabric‐skin interface during wearable use. Details of the experimental procedure are provided in Note S6 (Supporting Information). Under these conditions, the stretchable strain sensor exhibited inconsistent measurement results across repeated trials due to uncontrolled interfacial friction and slippage. The measured curvature deviated from the theoretical value by ≈46% (Figure 4b). In contrast, the developed sensor exhibited a deviation of only 2.9% under the same uncontrolled interfacial friction and slippage conditions (Figure 4c), achieving a 93.6% reduction in error. Another comparison with non‐stretchable curvature sensors^[^ ^21^ ^]^ involved evaluating large‐scale gestures, such as fist clenching, which typically induce joint strains of up to 10%. Owing to its low stiffness and high stretchability, the developed sensor accommodates such deformation reliably without wrinkling or detachment, in contrast to non‐stretchable curvature sensors (Figure S14, Supporting Information). The strain level on the finger surface during motions such as fist clenching was calculated to be ≈6%. Detailed information is provided in Note S7 (Supporting Information). Our overall design principle is to ensure adequate stretchability for the required strain range first, and then maximize the GF. Notably, its stiffness is ≈1/200 that of conventional non‐stretchable designs. This substantial reduction in stiffness, as detailed in Note S8 (Supporting Information), underlies the sensor's superior adaptability to large‐strain deformation. Therefore, while the GF of our sensor is lower than that reported for high‐GF strain sensors,^[^ ^32^, ^33^, ^34^ ^]^ our design delivers superior comprehensive performance‐linearity, robustness, and interference immunity‐which is more aligned with the core requirements of flexible robotics and wearable curvature sensing.

Figure 4d shows four distinct gestures and their corresponding real‐time curvature signals. To demonstrate the capabilities of the smart glove in human‐computer interaction, we employed gesture‐based commands to guide a hexapod robot through a maze (Figure 4e), with gestures one to four corresponding to turning left, moving forward, moving backward, and turning right, respectively (Figure 4f). Additionally, we demonstrated the capability for robotic manipulation by using hand gestures to remotely control a mechanical hand for grasping hazardous objects (Figure 4g), with the robotic hand performing corresponding movements in response to human gestures. The entire operation was divided into six sequential stages: hand elevation, hand opening, object seizing, hand rotation, object releasing, and hand relaxation. To assess the sensor's sensitivity to subtle motions, we conducted subtle motion‐distinction tests. The sensor‐integrated glove produced clearly distinguishable signals when grasping cylinders of 40, 35, and 30 mm, demonstrating sufficient resolution for fine bending variations in real‐world, clinical, and human‐robot applications. Furthermore, we performed multi‐condition durability tests on the sensor, including 45 min of exercise to induce natural sweating, 12 h of daily‐life wearing, and 1 h of water immersion. The results are shown in Figure S16 (Supporting Information). These results demonstrate the developed sensor's high measurement fidelity and mechanical adaptability. Such capabilities enable more effective human‐machine interaction and enhance the operational performance of disaster rescue robots in hazardous environments, including earthquakes, fires, and nuclear contamination.^[^ ^35^ ^]^

## 3 Conclusion

In summary, we report a stretchable curvature sensor based on the WSSLS with high repeatability, high linearity, and bending‐stretching decoupling, along with the curved surface lithography technique for fabricating 3D conductive foils free of initial stress. The structural design, sensing mechanism analysis, sensor fabrication, theoretical modeling, FEA of force‐electric characteristics, performance evaluation, and application demonstrations were systematically conducted. The collective results indicate that an appropriate ratio of the wave structure and thickness dimensionless parameters can be adopted to balance κ_e_, GF, and elastic range. Stretching and bending the sensor results in pure bending of the substrate, which directly leads to geometric transformation of the conductive foil, thereby ensuring high repeatability and linearity (*R* ^2^ > 0.999). The unique WSSLS imparts stretchability to the sensor while also enabling self‐compensation for pressure and temperature. The DSD mechanism enables effective decoupling of bending and stretching. Compared to both stretchable strain sensors and non‐stretchable curvature sensors, the stretchable curvature sensor delivers consistent measurements under friction and slippage, while also offering enhanced softness, improved comfort, and effective adaptation to large‐gesture deformations. These features enable reliable gesture recognition and precise device control. Leveraging these design concepts, the curved surface lithography technique and WSSLS frameworks can be further advanced through the integration of diverse functional materials, enabling an expanded spectrum of applications. Beyond these performance merits, the fabrication process employs mature and standardized techniques, including photolithography and magnetron sputtering, which are widely adopted in flexible electronics manufacturing. These methods are compatible with continuous roll‐to‐roll processing and therefore offer a feasible pathway toward large‐scale production with controllable wave morphology. Nevertheless, challenges such as interfacial delamination, yield rates, and real‐world process integration still need to be systematically addressed, and resolving these issues will be a central focus of our subsequent work.

## 4 Experimental Section

### Fabrication Process of the Curvature Sensors

The fabrication process of the curvature sensors is illustrated in Figure S1 (Supporting Information) and consists of the following steps: Step 1: The photoresist (AZ5214) was spin‐coated at 2000 rpm for 30 s onto a PE film (7 cm×7 cm×31 µm, Dalian, Liaoning, China), followed by prebaking at 110 C for 3 min. Prebaking was performed on the checkered cushion film. The details are provided in Note S9 (Supporting Information) and illustrated in Figure S17 (Supporting Information). The film was then exposed to UV light, developed, flipped, and the process was repeated to produce grooves on both sides, designed to accommodate conductive foils. Step 2: The film with conductive foil grooves on both sides was patterned by a UV picosecond laser (DL566PU, DCT, China). Step 3: The patterned film was placed in an aerospace‐grade aluminum mold and fastened with screws. Then, it was put into a 200  C oven for 20 min. As a result, the wave‐shaped film with conductive foil grooves on both sides was obtained. Step 4: Cr and Pt were magnetron‐sputtered on both sides of the film respectively. Step 5: WSSLS was obtained by ultrasonically removing excess photoresist and platinum using acetone. Step 6: Four copper wires were attached to the junction points of the WSSLS using conductive epoxy resin adhesive, serving as electrodes for external circuit connection. Step 7: The WSSLS with the copper wires was encapsulated with a 0.3 mm‐thick encapsulation layer (Dragon Skin 00–30, Smooth‐On, USA). An additional coating of a 1:1 liquid mixture of Dragon Skin was applied over the structure, followed by curing.

### Fabrication Process of the Smart Gloves

To ensure reliable adhesion of the curvature sensor on fabric‐based wearable substrates, conducted a comparative evaluation of three representative adhesion materials, including Dragon Skin, ergo, and Kafuter flexible adhesive. Static adhesion tests, dynamic bending cycles, and large‐strain deformation tests were performed to assess the long‐term stability and mechanical compatibility. Ergo exhibited high initial bonding strength but developed microcracks after curing due to its rigid structure, resulting in interfacial delamination under cyclic bending. Dragon Skin demonstrated limited bonding affinity to fabrics, leading to partial edge detachment under large curvature deformation. In contrast, Kafuter flexible adhesive maintained strong interfacial integrity and high flexibility after curing, showing no observable detachment or performance degradation after 2000 bending‐stretching cycles. Therefore, Kafuter flexible adhesive was selected as the optimal material for sensor integration on wearable platforms. Furthermore, the results of the Kafuter interface realistic wear‐condition adhesion test are shown in Figure S18 (Supporting Information). The sensors were attached to a soft, stretchable glove using an optically transparent adhesive (Kafuter flexible adhesive), with each sensor precisely aligned to the finger joints. These sensors conform closely to the fingers and can deform freely as the fingers bend. A serpentine flexible printed circuit board interconnected the sensors and connected to a data processing module positioned on the dorsal side of the wrist. This module integrates a data processing chip and a Bluetooth module. The data processing chip analyzes resistance values to identify gesture types and transmits corresponding Bluetooth signals to control the robot and mechanical hand accordingly.

### Ethical Approval and Written Consent

All procedures involving human participants were conducted in accordance with the ethical standards of the institutional and/or national research committee and with the 1964 Helsinki Declaration and its later amendments or comparable ethical standards. The study was approved by the Institutional Review Board of the Institute of Mechanics, Chinese Academy of Sciences (Approval No. 20 240 103). Written informed consent was obtained from all participants prior to their involvement in the study.

### Characterization of the Curvature Sensors

Static, dynamic, and cyclic tests of the stretchable curvature sensors were carried out by a programmable stretch testing machine (42 Universal Testing Machine, MTS Systems Corporation, USA), as shown in Figure S19 (Supporting Information). The resistance and voltage signals of the sensors were measured by a digital multimeter (34461A, Keysight, USA). The curvature sensor with pressure and temperature self‐compensation was powered by a programmable DC power (DP831A, RIGOL, China).

### Finite Element Analyses of Deformation

The FEA was performed employing the commercial software ABAQUS (SIMULIA, France) to verify the mechanical model of the WSSLS. A half period of the WSSLS was used for the FEA model, with the boundary conditions of zero displacement imposed in the x direction at one end cross section. A displacement load of L and a bending moment load of M were applied in the same direction at another end cross section. PE (elastic modulus = 0.8 GPa and Poisson's ratio = 0.42) and Pt (elastic modulus = 168 GPa and Poisson's ratio = 0.38) were both regarded as linear elastic materials. The hexahedron element C3D8R was utilized for PE and Pt. The deformation result of the FEA agreed well with that of the experiment, as shown in Figure S20 (Supporting Information).

### Finite Element Analyses of the Change in Resistance

The model of resistance variation of the WSSLS was solved by FEA with the commercial software COMSOL. The steady‐state research was carried out by choosing the physical field “solid mechanics”. The mechanical boundary conditions, the elastic modulus, and Poisson's ratio were the same as those in ABAQUS. Pt's resistivity is 105 nΩ m. By measuring the line lengths of the top and bottom surfaces and the angles at both ends, and substituting these values into Equation S10 (Supporting Information), a finite element–based relationship between electrical response and curvature was established.

## Conflict of Interest

The authors declare no conflict of interest.

## Author Contributions

Y.S. and T.W. conceived the concept. T.W. conducted all the experimental fabrication and performance testing. T.W., X.X., S.L., Y.L, K.C., G.W., K.Y., L.S., and Y.S. discussed all the data and contributed to the manuscript preparation. Y.S. supervised the project.

## Supporting information



Supporting Information

## Data Availability

The data that support the findings of this study are available from the corresponding author upon reasonable request.

## References

[1] S. Pyo , J. Lee , K. Bae , S. Sim , J. Kim , Adv. Mater. 2021, 33, 2005902. 10.1002/adma.202005902 33887803

[2] M. Liao , P. Wan , J. Wen , M. Gong , X. Wu , Y. Wang , R. Shi , L. Zhang , Adv. Funct. Mater. 2017, 27, 1703852.

[3] Y. R. Jeong , H. Park , S. W. Jin , S. Y. Hong , S.‐S. Lee , J. S. Ha , Adv. Funct. Mater. 2015, 25, 4228.

[4] G. Shi , Z. Zhao , J.‐H. Pai , I. Lee , L. Zhang , C. Stevenson , K. Ishara , R. Zhang , H. Zhu , J. Ma , Adv. Funct. Mater. 2016, 26, 7614.

[5] G. Ge , Y. Zhang , J. Shao , W. Wang , W. Si , W. Huang , X. Dong , Adv. Funct. Mater. 2018, 28, 1802576.

[6] M. Iskandar , A. Albu‐Schaeffer , A. Dietrich , Sci. Rob. 2024, 9. 10.1126/scirobotics.adn4008 39167671

[7] H. Zhao , K. O'Brien , S. Li , R. F. Shepherd , Sci. Rob. 2016, 1, aai7529. 10.1126/scirobotics.aai7529 33157858

[8] S. N. Flesher , J. E. Downey , J. M. Weiss , C. L. Hughes , A. J. Herrera , E. C. Tyler‐Kabara , M. L. Boninger , J. L. Collinger , R. A. Gaunt , Science 2021, 372, 831. 34016775 10.1126/science.abd0380 PMC8715714

[9] R. Bao , J. Tao , J. Zhao , M. Dong , J. Li , C. Pan , Sci. Bull. 2023, 68, 1027. 10.1016/j.scib.2023.04.019 37120379

[10] S. He , J. Dai , D. Wan , S. Sun , X. Yang , X. Xia , Y. Zi , Sci. Adv. 2024, 10, ado6793. 10.1126/sciadv.ado6793 PMC11225791 38968360

[11] Y. Qiu , F. Wang , Z. Zhang , K. Shi , Y. Song , J. Lu , M. Xu , M. Qian , W. Zhang , J. Wu , Z. Zhang , H. Chai , A. Liu , H. Jiang , H. Wu , Sci. Adv. 2024, 10, adp0348. 10.1126/sciadv.adp0348 PMC11268415 39047112

[12] Q. Su , Q. Zou , Y. Li , Y. Chen , S.‐Y. Teng , J. T. Kelleher , R. Nith , P. Cheng , N. Li , W. Liu , S. Dai , Y. Liu , A. Mazursky , J. Xu , L. Jin , P. Lopes , S. Wang , Sci. Adv. 2021, 7, abi4563. 10.1126/sciadv.abi4563 PMC8612682 34818045

[13] S. Raspopovic , G. Valle , F. M. Petrini , Nat. Mater. 2021, 20, 925. 33859381 10.1038/s41563-021-00966-9

[14] Q. He , Z. Wang , Y. Wang , A. Minori , M. T. Tolley , S. Cai , Sci. Adv. 2019, 5, aax5746. 10.1126/sciadv.aax5746 PMC6788870 31646178

[15] J. Ren , Y. Liu , Z. Wang , S. Chen , Y. Ma , H. Wei , S. Lü , Adv. Funct. Mater. 2022, 32, 2107404.

[16] Y. Bu , T. Shen , W. Yang , S. Yang , Y. Zhao , H. Liu , Y. Zheng , C. Liu , C. Shen , Sci. Bull. 2021, 66, 1849. 10.1016/j.scib.2021.04.041 36654394

[17] T. Yamada , Y. Hayamizu , Y. Yamamoto , Y. Yomogida , A. Izadi‐Najafabadi , D. N. Futaba , K. Hata , Nat. Nanotechnol. 2011, 6, 296. 21441912 10.1038/nnano.2011.36

[18] H. Wang , R. Zhou , D. Li , L. Zhang , G. Ren , L. Wang , J. Liu , D. Wang , Z. Tang , G. Lu , G. Sun , H.‐D. Yu , W. Huang , ACS Nano 2021, 15, 9690. 34086439 10.1021/acsnano.1c00259

[19] H. Chen , F. Zhuo , J. Zhou , Y. Liu , J. Zhang , S. Dong , X. Liu , A. Elmarakbi , H. Duan , Y. Fu , Chem. Eng. J. 2023, 464, 142576.

[20] S. Li , G. Liu , R. Li , Q. Li , Y. Zhao , M. Huang , M. Zhang , S. Yin , Y. Zhou , H. Tang , L. Wang , G. Fang , Y. Su , ACS Nano 2022, 16, 541. 34919398 10.1021/acsnano.1c07645

[21] H. Liu , H. Zhao , S. Li , J. Hu , X. Zheng , R. Li , Y. Chen , Y. Su , Adv. Mater. Technol. 2019, 4, 1800327.

[22] O. A. Araromi , M. A. Graule , K. L. Dorsey , S. Castellanos , J. R. Foster , W.‐H. Hsu , A. E. Passy , J. J. Vlassak , J. C. Weaver , C. J. Walsh , R. J. Wood , Nature 2020, 587, 219. 33177670 10.1038/s41586-020-2892-6

[23] C. Yan , X. Li , X. Wang , G. Liu , Z. Yang , H. Tian , C. Wang , X. Chen , J. Shao , Adv. Funct. Mater. 2024, 34, 2409093.

[24] H. Luan , Q. Zhang , T.‐L. Liu , X. Wang , S. Zhao , H. Wang , S. Yao , Y. Xue , J. W. Kwak , W. Bai , Y. Xu , M. Han , K. Li , Z. Li , X. Ni , J. Ye , D. Choi , Q. Yang , J.‐H. Kim , S. Li , S. Chen , C. Wu , D. Lu , J.‐K. Chang , Z. Xie , Y. Huang , J. A. Rogers , Sci. Adv. 2021, 7.

[25] S. Ryu , P. Lee , J. B. Chou , R. Xu , R. Zhao , A. J. Hart , S.‐G. Kim , ACS Nano 2015, 9, 5929. 26038807 10.1021/acsnano.5b00599

[26] J.‐H. Lee , H. Chen , E. Kim , H. Zhang , K. Wu , H. Zhang , X.i Shen , Q. Zheng , J. Yang , S. Jeon , J.‐K. Kim , Mater. Horiz. 2021, 8, 1488. 34846457 10.1039/d1mh00018g

[27] C. Wang , X. Li , E. Gao , M. Jian , K. Xia , Q. Wang , Z. Xu , T. Ren , Y. Zhang , Adv. Mater. 2016, 28, 6639. 27511531 10.1002/adma.201670215

[28] M. Hempel , D. Nezich , J. Kong , M. Hofmann , Nano Lett. 2012, 12, 5714. 23045955 10.1021/nl302959a

[29] H. Bai , S. Li , J. Barreiros , Y. Tu , C. R. Pollock , R. F. Shepherd , Science 2020, 370, 848. 33184214 10.1126/science.aba5504

[30] Y. Su , J. Wu , Z. Fan , K.‐C. Hwang , J. Song , Y. Huang , J. A. Rogers , J. Mech. Phys. Solids 2012, 60, 487.

[31] S. Timoshenko , J. Gere , W. Prager , J. Appl. Mech. 1961, 29, 220.

[32] Q. H. Yu , R. Ge , J. Wen , T. Du , J. Zhai , S. Liu , L. Wang , Y. Qin , Nat. Commun. 2022, 13. 10.1038/s41467-022-28443-0 PMC8828782 35140219

[33] R. Madhavan , New J. Chem. 2025, 49, 1700.

[34] C. Pang , G.‐Y. Lee , T.‐I. Kim , S. M. Kim , H. N. Kim , S.‐H. Ahn , K.‐Y. Suh , Nat. Mater. 2012, 11, 795. 22842511 10.1038/nmat3380

[35] Y. Ni , X. Zang , J. Chen , T. Zhu , Y. Yang , J. Huang , W. Cai , Y. Lai , Adv. Funct. Mater. 2023, 33, 2301127.

