# Supplementary material for: Stretchable Curvature Sensors for Motion Capture with Bending‐Stretching Coupling Deformation
Source: Adv Sci (Weinh). 2025 Dec 16;13(12):e14779. doi: 10.1002/advs.202514779 (PMC12948215; doi:10.1002/advs.202514779)
Supplement: Supplementary file 1 — Supporting Information [file ADVS-13-e14779-s001.docx]

Supplementary Materials for

Stretchable curvature sensors for motion capture with bending-stretching coupling deformation

Tairan Wang,^1,2^ Xinkai Xu,^1,2^ Shuang Li,^1,3^ Yuqun Lan,^1,2^ Kai Chen,^1,2^ Wei Li,^1,5^ Guangyuan Wang,^4^ Kuai Yu,^4^ Lijuan Sun,^1,2^ Yewang Su, ^1,2,5,6,*^

*^1^ State Key Laboratory of Nonlinear Mechanics, Institute of Mechanics, Chinese Academy of Sciences, Beijing 100190, China*

*^2^ School of Engineering Science, University of Chinese Academy of Sciences, Beijing 100049, China*

*^3^ Institute of Biomechanics and Medical Engineering, AML, Department of Engineering Mechanics, Tsinghua University, Beijing 100084, China*

*^4^ China Academy of Space Technology, Haidian District, Beijing, PR China, 100080*

*^5^ State Key Laboratory of Intelligent Manufacturing Equipment and Technology, Huazhong University of Science and Technology, Wuhan 430074, China*

*^6^ Zhongke Technology Achievement Transfer and Transformation Center of Henan Province, Changyuan County, Henan 453000, China*

^*^To whom all correspondence should be addressed: [yewangsu@imech.ac.cn](mailto:yewangsu@imech.ac.cn)

Supplementary notes

**Note S1. Derivation of the relationship between the resistance variation of the conductive foil and the relative variation in output voltage of the Wheatstone bridge.**

To quantify the relationship between the resistance change of the conductive foil and the relative variation in output voltage, we analyze the behavior of a Wheatstone bridge incorporating the conductive foil as a sensitive element, as shown in Figure S2. The bridge consists of four resistive arms, with two resistors ($R_{1}$ and $R_{3}$) undergoing resistance changes under mechanical deformation. The total current in the Wheatstone bridge ($I_{\text{total}}$) circuit is governed by Kirchhoff’s laws and can be expressed as:

$$I_{\text{total}}=\frac{U}{R_{_{\text{total}}}}=\frac{U}{\frac{(R_{1}+R_{3})(R_{2}+R_{4})}{R_{1}+R_{2}+R_{3}+R_{4}}}$$

where $U$ is the input voltage, $R_{\text{total}}$ is the total resistance of the Wheatstone bridge. $R_{2}$ and $R_{4}$ are fixed resistors, and their resistance values are equal to $R_{\text{s}}$. Using the current division rule, the current $I_{1}$ through one branch of the Wheatstone bridge is given by:

$$I_{1}=\frac{(R_{2}+R_{4})}{R_{1}+R_{2}+R_{3}+R_{4}}I_{_{\text{total}}}=\frac{U}{(R_{1}+R_{3})}$$

The calculation for the current in the opposite branch ($I_{2}$) follows the same approach and yields:

$$I_{2}=\frac{U}{(R_{2}+R_{4})}$$

Subsequently, we analyze the balance state of the Wheatstone bridge after deformation. In the initial state, both the top and bottom conductive foils are strain-free, with identical resistance denoted as $R$. When the sensor undergoes bending, the top conductive foil is subjected to tensile strain, while the bottom conductive foil experiences compressive strain. The resistance variations of the two conductive foils are identical, each denoted as $\Delta R$. The resistances of the two foils change to ($R_{1}=R+\Delta R$) and ($R_{3}=R-\Delta R$), respectively. The electrical potential difference between points a and b ($\Delta U$) can be expressed as:

where and are the electrical potential of points a and b. From Eq. S4, we obtain the relative variation in output voltage of the Wheatstone bridge:

$$\frac{\Delta U}{U}=\frac{\Delta R}{2R}$$

**Note S2. Derivation of the quantitative relationship between curvature and resistance variation.**

To establish the relationship between the curvature and the resistance change of the conductive foil, we begin with the mechanical analysis of a unit cell in the wave-shaped symmetrical laminated structure (WSSLS), subjected to a bending moment $M$, as shown in Figure S3a. The WSSLS consists of upper and lower Pt and PE layers (bottom-left subfigure of Figure S3a). As the Pt layer is only 50 nm thick, which is negligible relative to the 31 μm-thick PE layer, its mechanical influence on the structure is minimal and is therefore excluded from the analysis.

Consider an infinitesimal differential element of the PE layer. According to classical beam theory, the strain distribution across the thickness is linear, and the surface strain of the PE layer $\varepsilon$ is related to the curvature $\kappa$ by:

$$\varepsilon=\frac{h}{2}\kappa$$

Here, $h$ is the total thickness of the PE layer. From the geometric configuration in bottom-right subfigure of Figure S3a, the local curvature $\kappa$ can be expressed in terms of the rotation angle $\text{d}\theta$ of the differential element and its arc length $\text{d}S$:

$$\text{d}\theta=\kappa\text{d}S$$

Simultaneous substitution and solution of Eqs. S6 and S7 yield:

$$\text{d}\theta=\frac{2\varepsilon}{h}\text{d}S$$

By integrating both sides of Eq. S8 with respect to the arc length along the unit cell, we obtain:

$$\frac{\theta}{S}=\frac{2\varepsilon}{h}$$

where $\theta$ denotes the total rotation angle and $S$ represents the arc length of the unit cell. To connect strain to resistance, we invoke the strain-resistance relationship for the conductive foil:

$$\frac{\Delta R}{R}=(1+2\mu)\varepsilon$$

where $\mu$ is the Poisson’s ratio of the conductive material. By combining Eqs. S9 and S10, we obtain:

$$\theta=\frac{\Delta R}{R}\frac{2S}{h(1+2\mu)}$$

Next, consider the macroscopic deformation of an encapsulated unit, where $L$ denotes the length of a single encapsulated unit cell, and $\kappa$ represents its curvature, as illustrated in Figure S3b. From geometric relationships, we have:

$$\kappa=\frac{\theta}{L}$$

Substituting Eq. S12 into Eq. S11 yields the final relation between curvature and normalized resistance change:

$$\kappa=\frac{\Delta R}{R}\frac{2S}{(1+2\mu)Lh}$$

This establishes a direct link between the macroscopic curvature of the sensor and the relative resistance variation of the conductive foil, forming the theoretical foundation for curvature sensing.

**Note S3. Derivation of the analytical relationship between gauge factor and strain.**

According to Eq. 5, it can be derived that the GF is proportional to the $L$, during the stretching process, $L=L_{0}(1+\varepsilon)$, where $L_{0}$ is the original length. Therefore, the equations of GF and strain ($\varepsilon$) are expressed as follows:

$$\text{GF}=\frac{\text{d}}{\text{d}\kappa}\left(\frac{\Delta U}{U}\right)=\frac{(1+2\mu)hL_{0}(1+\varepsilon)}{4S}$$

:

**Note S4. Derivation of the curvature limit** $\kappa_{\text{e}}$**.**

To determine the effective curvature range of the stretchable curvature sensor, we derive the maximum elastic curvature. The curvature of the WSSLS is defined as $\kappa_{\text{e}}$ at the onset of plastic deformation in the conductive foil. A simultaneous solution of Eqs. S10 and S13 leads to:

$$\kappa=\frac{2S\varepsilon}{Lh}$$

To avoid plastic deformation, the strain of the conductive foils ($\varepsilon$) must not exceed the material’s elastic limit strain. $\varepsilon_{\text{Pt,max}}$ denotes the elastic limit strain of platinum. Therefore, the limiting curvature $\kappa_{\text{e}}$ is the curvature at which $\varepsilon=\varepsilon_{\text{Pt,max}}$, yielding:

$$\kappa_{\text{e}}=\frac{2S\varepsilon_{\text{Pt,max}}}{Lh}$$

This expression defines the upper bound of the sensor’s operating curvature range, beyond which the conductive foil may enter the plastic regime, leading to irreversible changes and measurement degradation. The elastic limit strain of platinum is as follows:

$$\varepsilon_{\text{Pt,max}}\approx0.5\%$$

**Note S5. Experimental procedure for the slip-simulation tests.**

To demonstrate the structural stability from a practical perspective, we conducted long-term durability testing and stability evaluations under varying environmental conditions.

(a) Long-term durability testing

Considering real-world wearable applications, the sensor is expected to endure extensive finger bending and unavoidable sweat exposure during prolonged use. To evaluate the long-term mechanical durability under repeated finger-bending deformation, we have already performed cyclic bending tests exceeding 2000 loading cycles, as presented in the main text. The results confirm that the device maintains a stable signal response without performance degradation.

In addition, to simulate sweat and humid conditions that are inevitable during long-term wear, we conducted a 24-hour liquid immersion test in deionized water, serving as an accelerated test for prolonged moisture exposure, which represents a more stringent condition than typical real-use scenarios. As shown in Figure S8a, the post-test curvature response remained nearly identical to the pre-test behavior, demonstrating excellent durability and moisture resistance.

(b) Stability tests under varying environmental conditions

To further evaluate environmental stability, the sensor was exposed to low-temperature (4 ℃) and high-temperature (50 ℃) conditions for 24 hours, simulating operation in cold and hot climates, respectively. As illustrated in Figures S8b and 8c, negligible deviation was observed in the response curves before and after testing, confirming that the sensor maintains high structural stability across a wide range of environmental conditions.

Overall, these results collectively verify that the proposed structure exhibits outstanding long-term durability and environmental stability, ensuring reliable operation under realistic wearable scenarios.

**Note S6. Experimental procedure for the slip-simulation tests.**

The developed sensor and a stretchable strain sensor were independently attached to a fabric substrate. The resulting sensor-integrated fabric was placed on a flat layer of soft silicone (Ecoflex 00-30, Smooth-On, USA) and gradually bent over an acrylic cylinder with a diameter of 80 mm, as shown in Figure S11. The uncontrolled interfacial slippage and friction between the fabric and the Ecoflex effectively simulates the unpredictable contact behavior at the fabric-skin interface during wearable use. Each experiment was repeated six times for each type of sensor. Between trials, the fabric was lifted and repositioned on the soft silicone to reset slippage and friction conditions.

**Note S7. Derivation of Strain Level in a Smart Glove.**

During the experiments, the relationship between the resistance change and the applied strain of the stretchable strain sensor was calibrated through uniaxial tensile testing. Based on the obtained calibration curve (Figure S12a), the corresponding strain levels during different gesture recognition tests were derived from the measured resistance variations (Figure S12b). The results indicate that the maximum strain experienced by the sensor during finger bending is approximately 6%, which is significantly lower than the elastic limit of the curvature sensor. This demonstrates that the device consistently operates within the safe elastic range throughout the entire range of gesture motions.

**Note S8. Comparative analysis of bending stiffness in curvature sensors.**

To quantitatively evaluate the mechanical compliance of the stretchable curvature sensor developed in this study, we conducted a comparative analysis of the bending stiffness between the proposed wavy symmetric stacked layer structure (WSSLS) and a traditional non-stretchable curvature sensor using finite element analysis (FEA).

The conventional non-stretchable curvature sensor was modeled as a 100 μm-thick planar polyimide (PI) substrate with a Young’s modulus of 4 GPa and a Poisson’s ratio of 0.38. Although the sensor also contains a 4 μm-thick constantan conductive layer, the metallic layer is much thinner than the substrate and contributes negligibly to the overall bending stiffness. Therefore, it was omitted from the FEA.

In contrast, the stretchable curvature sensor utilizes a WSSLS design composed of a 31 μm-thick polyethylene (PE) substrate with a Young’s modulus of 0.8 GPa and a Poisson’s ratio of 0.42. The structure features a periodic wavy geometry.

FEA was conducted using ABAQUS. To ensure consistency, both models were subjected to identical boundary conditions and loaded with a bending moment of 0.1 N·mm. The total rotational angle $\theta$ at the free end of the structure was recorded in each case. According to classical beam theory, the effective bending stiffness $D$ is defined as:

$$D=\frac{M}{\theta}$$

where $D$ is the bending stiffness, $M$ is the applied moment, and $\theta$ is the resulting total rotation angle.

Simulation results reveal that, under the same loading conditions, the rotational angle of the WSSLS sensor is significantly larger than that of the conventional design, indicating a much lower bending stiffness. Quantitative analysis shows that the effective bending stiffness of the developed stretchable curvature sensor based on the WSSLS is approximately 1/223 that of the non-stretchable curvature sensor. Detailed simulation results are presented in Supplementary Figure S13.

**Note S9. Detailed elaboration of the pre-baking of the checkered cushion film.**

In this study, the fabrication of the WSSLS requires sequential photolithography on both sides of a 31 μm thick polyethylene film substrate. Hence, it is necessary to optimize the pre-baking scheme to ensure: (1) the complete evaporation of the photoresist solvent to avoid issues such as flow, warping, and bubble formation during exposure; (2) the prevention of photoresist crosslinking on the first side during second side photolithography due to heat exposure.

Depending on the heating method, photoresist baking mainly includes hot plate baking and oven baking, each having its own advantages and disadvantages. Hot plate baking, although providing uniform heating and rapid solvent evaporation, is suitable for high-resolution lithography. However, it is not suitable for double-sided lithography, as the photoresist on the first side may crosslink during the second-side lithography, affecting subsequent development. Oven baking, although suitable for double-sided lithography and avoiding crosslinking issues, has a slower solvent evaporation rate, which may lead to a decrease in pattern resolution and difficulty in ensuring double-sided alignment accuracy.

To address the above-mentioned issues, this study proposes a pre-baking scheme for the checkered cushion film. That is, during hot plate baking, a 200 μm thick polyimide (PI) cross-shaped spacer film is placed between the hot plate and the substrate to achieve indirect heating. The polyimide cross-shaped spacer film adopts a large-area hollow structure to ensure rapid heat transfer to the photoresist and improve solvent evaporation efficiency. Meanwhile, the 200 μm thickness effectively isolates direct thermal contact, prevents overheating, and maintains good thermal conductivity. A comparative analysis of the advantages and disadvantages of the pre-baking method using the checkered cushion film and traditional methods is summarized in Table S2.

**Table S1. Direct benchmarking metrics comparing the proposed stretchable curvature sensor with representative flexible sensor technologies.**

| Sensor type | Stretchability | Bending-stretching decoupling | Linearity | Repeatability | Key limitations |
| --- | --- | --- | --- | --- | --- |
| This work | ✔ | ✔ | High | High | — |
| Single-sided wavy metal-foil sensor^[2]^ | ✔ | × | Moderate | Moderate | Coupling-induced nonlinearity; signal drift during stretching |
| Planar laminated metal-foil curvature sensor^[3]^ | × | ✔ | High | High | Intrinsically non-stretchable; poor wearable comfort |
| Stretchable strain sensor^[4]^ | ✔ | × | High | High | Severe bending-stretching coupling, leading to unreliable curvature measurements |
| Optical fiber / waveguide curvature sensor^[5]^ | ✔ | × | Moderate | Moderate | High stiffness; unsuitable for wearable applications |

**Table S2. Comparative analysis of the merits and demerits between the pre-baking of the checkered cushion film and traditional methods.**

| Parameter | Hot Plate Baking | Oven Baking | Pre-baking of the checkered cushion film |
| --- | --- | --- | --- |
| Heating Speed | Fast | Slow | Moderate |
| Solvent Evaporation Rate | Fast but Inhomogeneous | Uniform but Slow | Fast and Uniform |
| Substrate Deformation | May Occur | May Occur | Minimum Deformation |
| Suitable for Double-sided Lithography | No | Yes | Yes |
| Photoresist Crosslinking | Affects the Previous Layer of Photoresist | No Effect | No Effect |
| Resolution | High | Low | High |


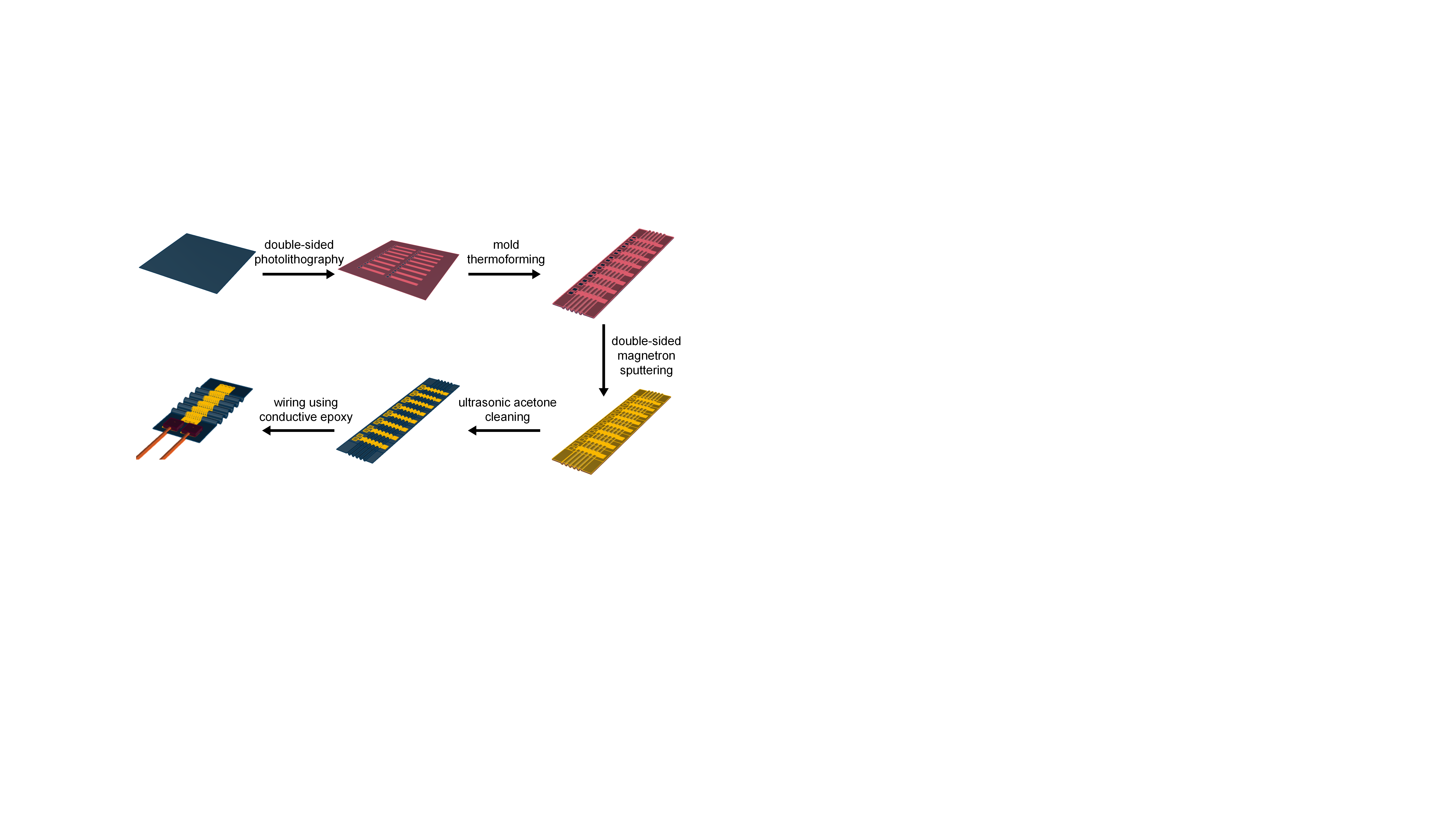


**Figure S1.** **The derivation process of the stretchable curvature sensor.**


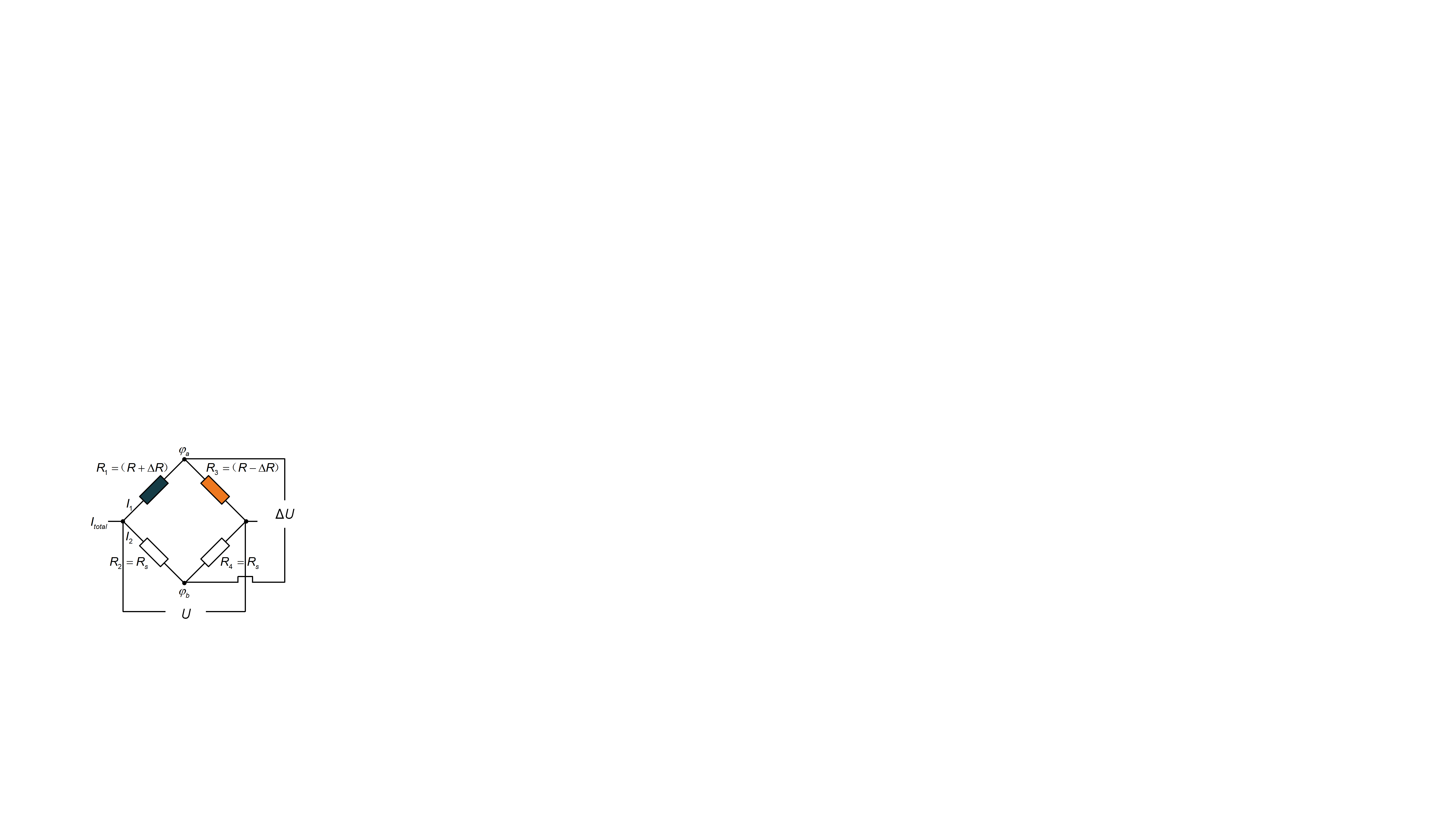


**Figure S2.** **Schematic of the derivation of the relationship between resistance variation and output voltage.**


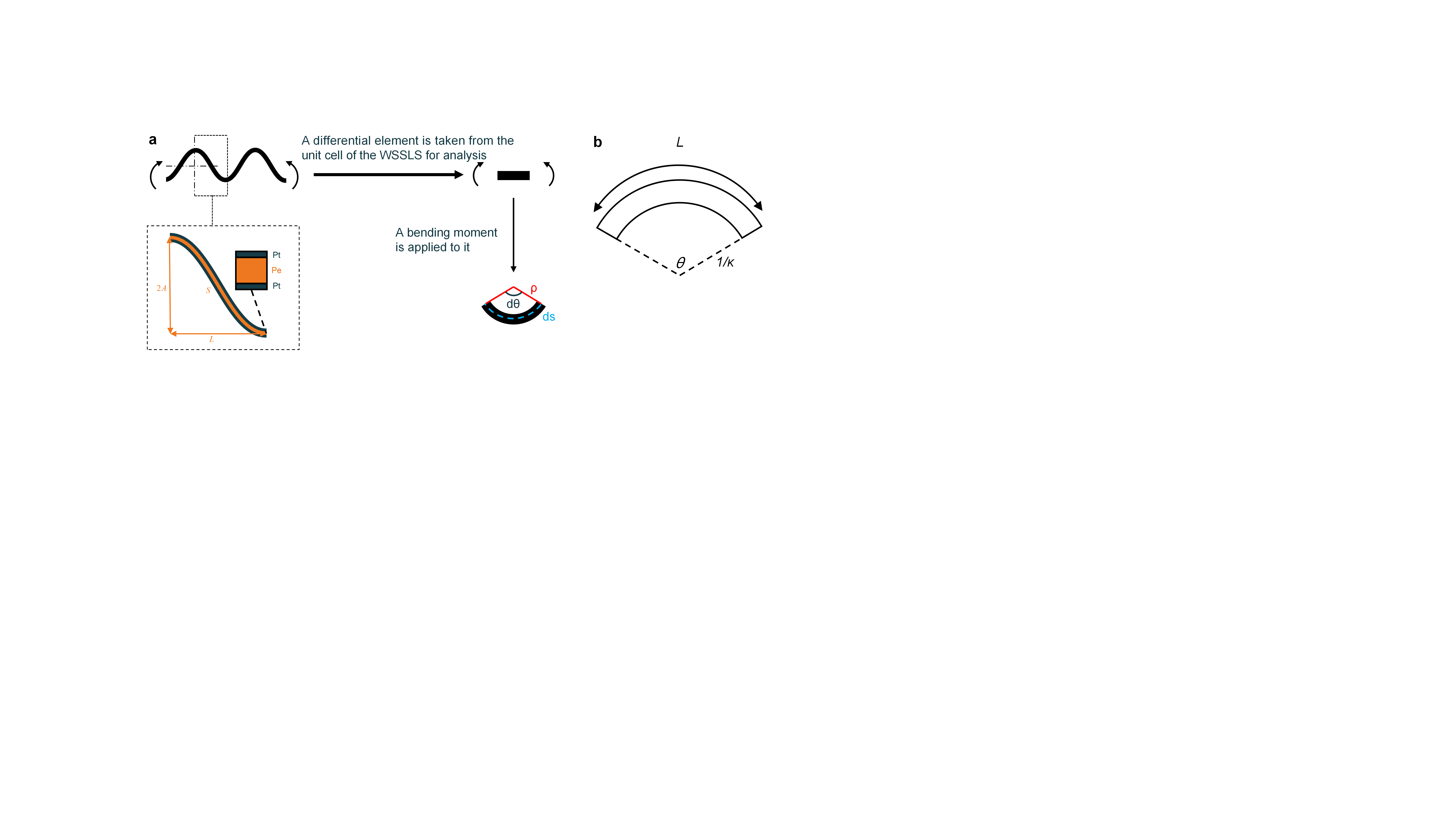


**Figure S3.** **Schematic of the derivation of the relationship between curvature and resistance variation.** **(a)** Geometry of a unit cell in the WSSLS. **(b)** Geometry of a single encapsulated unit cell.


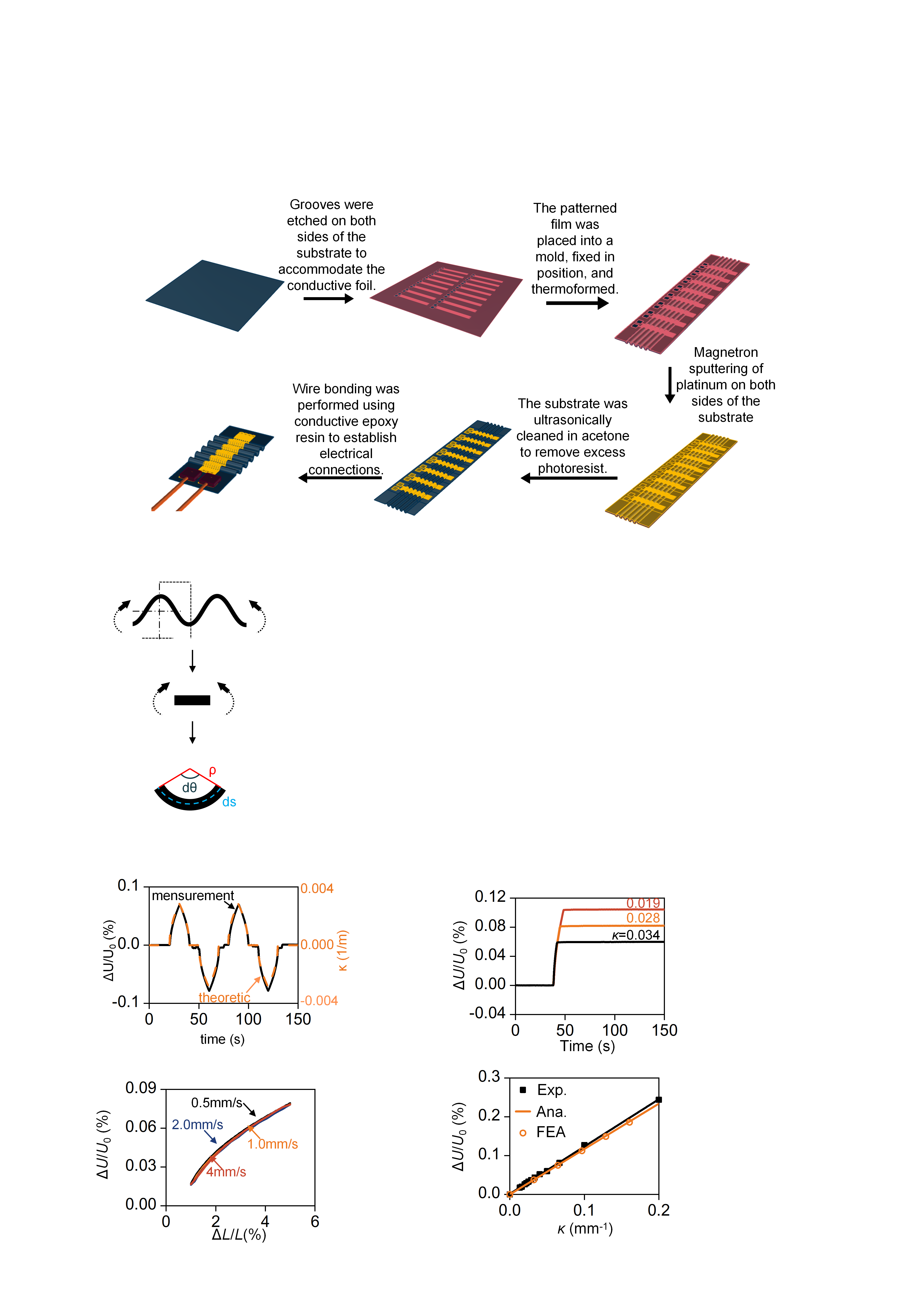


**Figure S4.** **Diagrams of experimental data for positive and negative curvature measurement.**


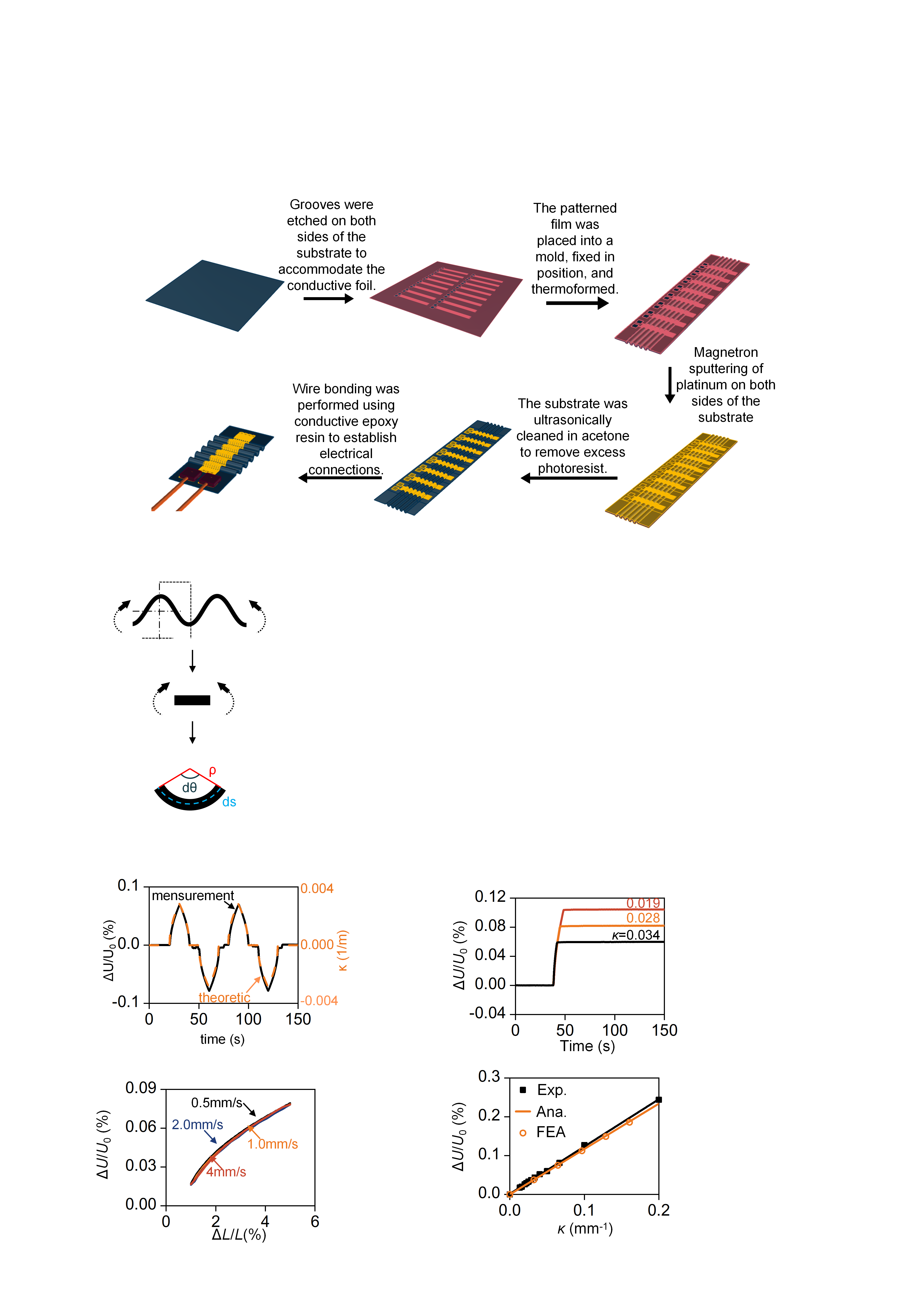


**Figure S5.** **Diagrams of experimental data demonstrating stability under different loading rates.**





**Figure S6.** **Extended-cycle fatigue test up to 8000 repetitions. The sensor performance at each 2000-cycle interval is presented separately.**


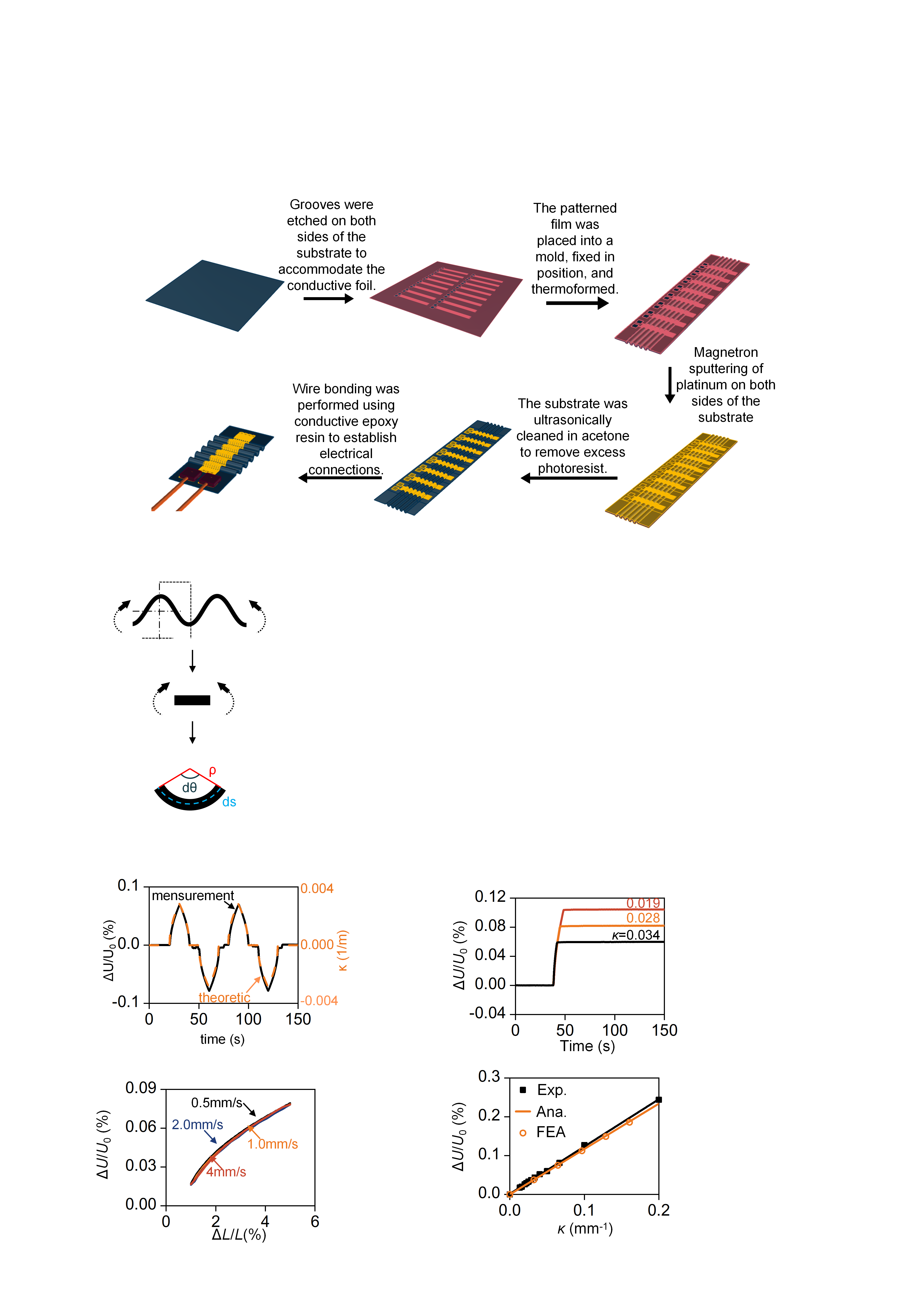


**Figure S7.** **Diagrams of experimental data under varying curvature loads.**


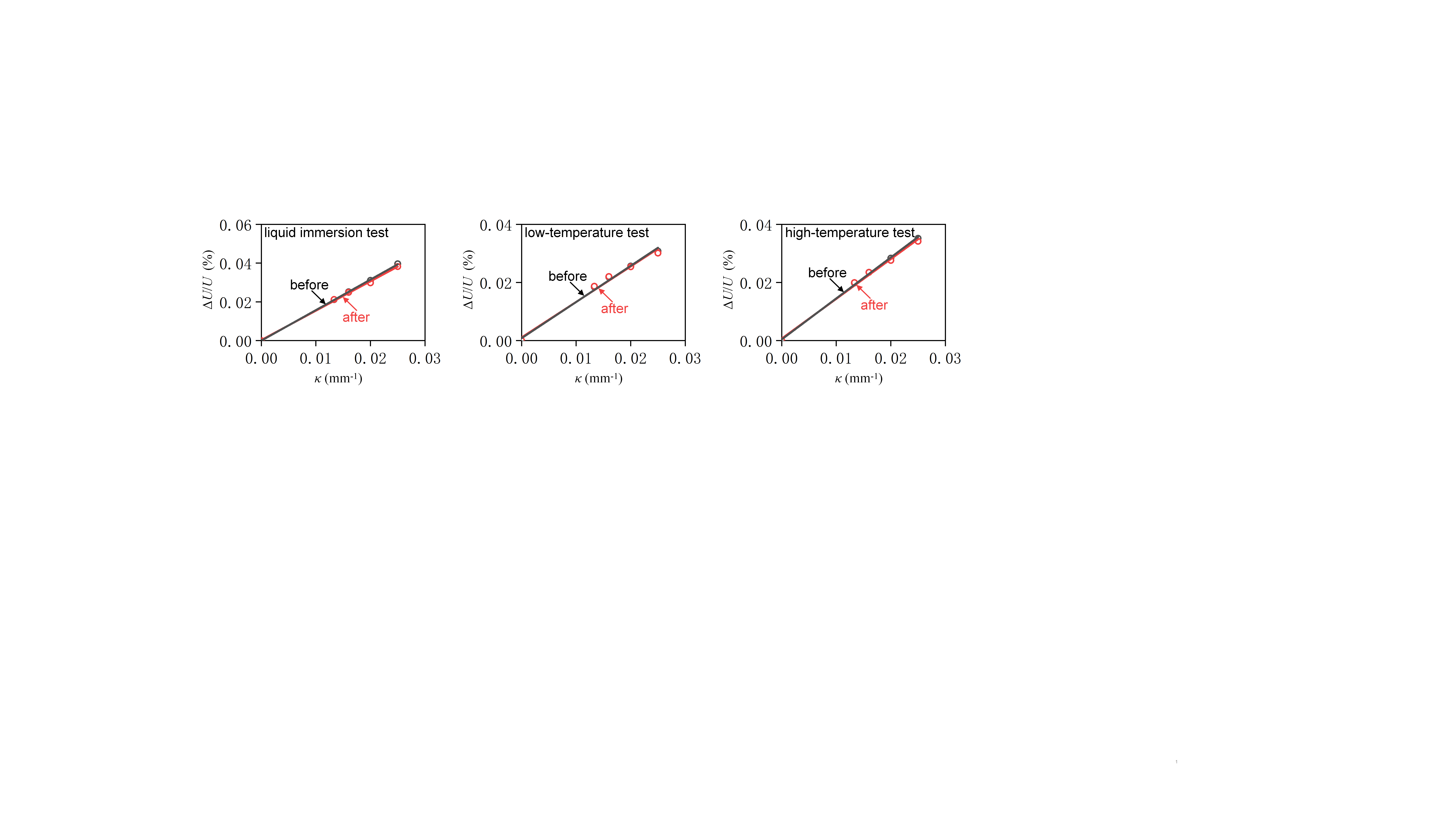


**Figure S8.** **Data of long-term durability testing under varying environmental conditions. (a) Liquid immersion test. (b) Low-temperature test. (c) High-temperature test.**


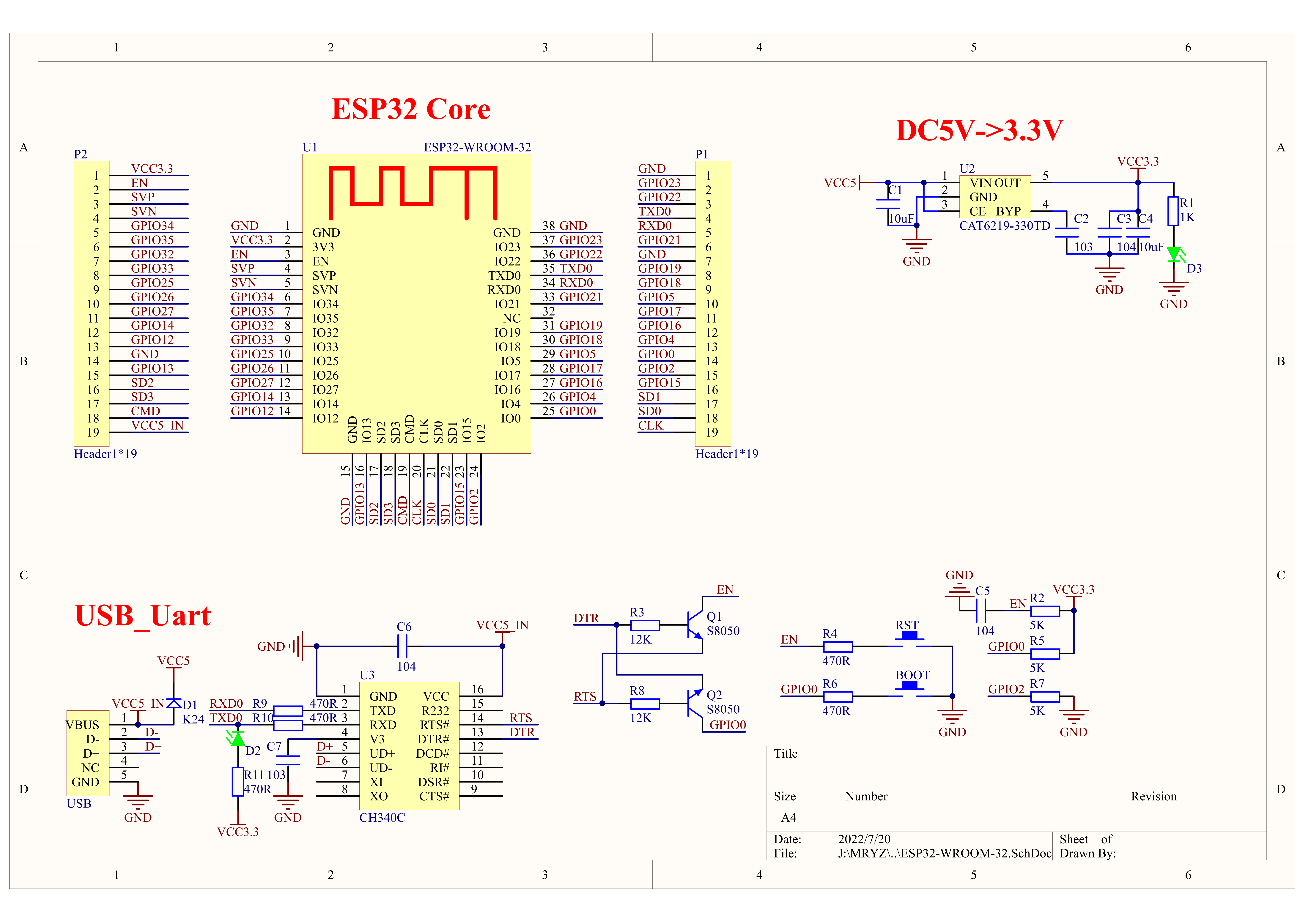


**Figure S9.** **Schematic diagram of the core board.**


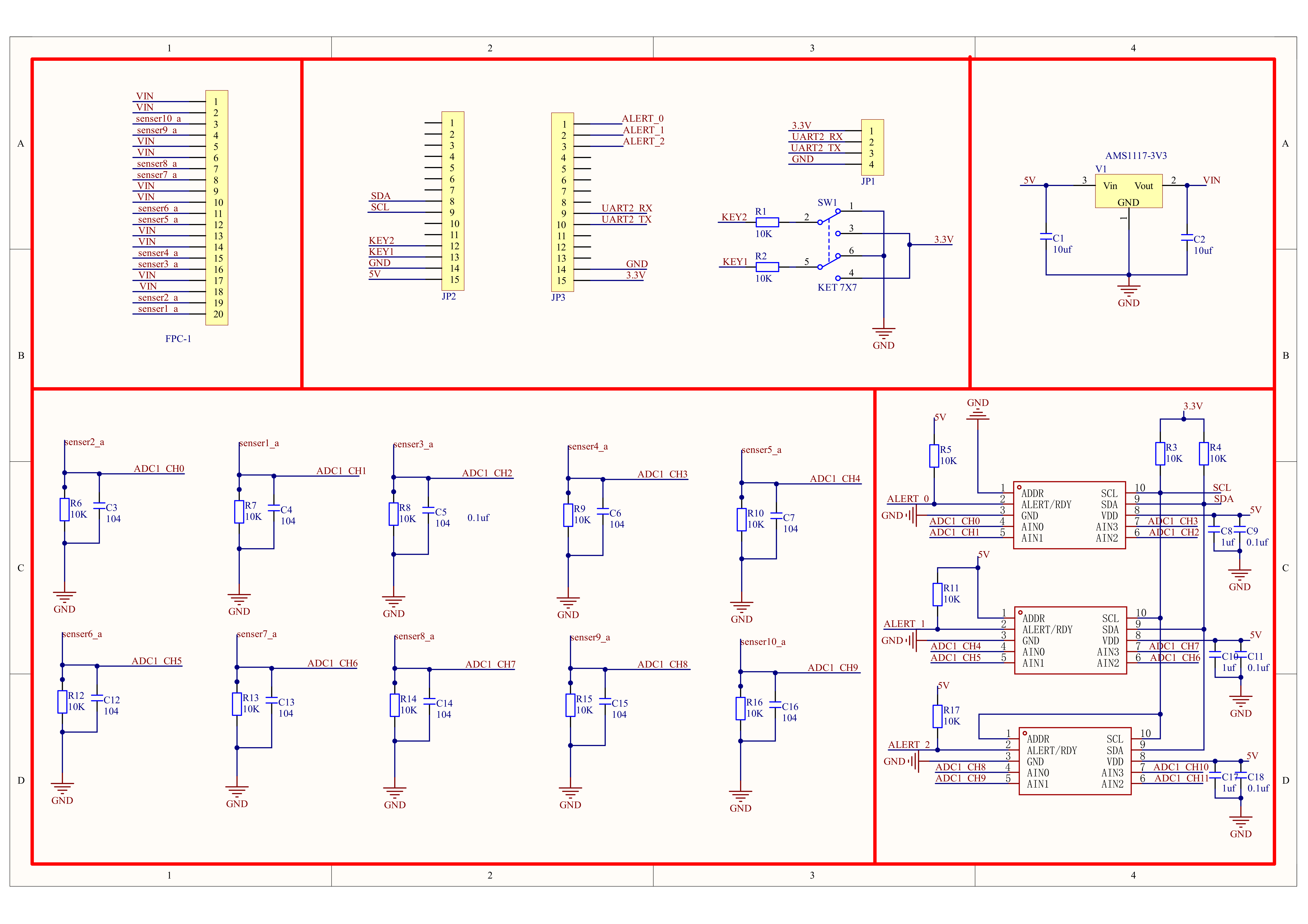


**Figure S10.** **Schematic diagram of the bottom plate .**


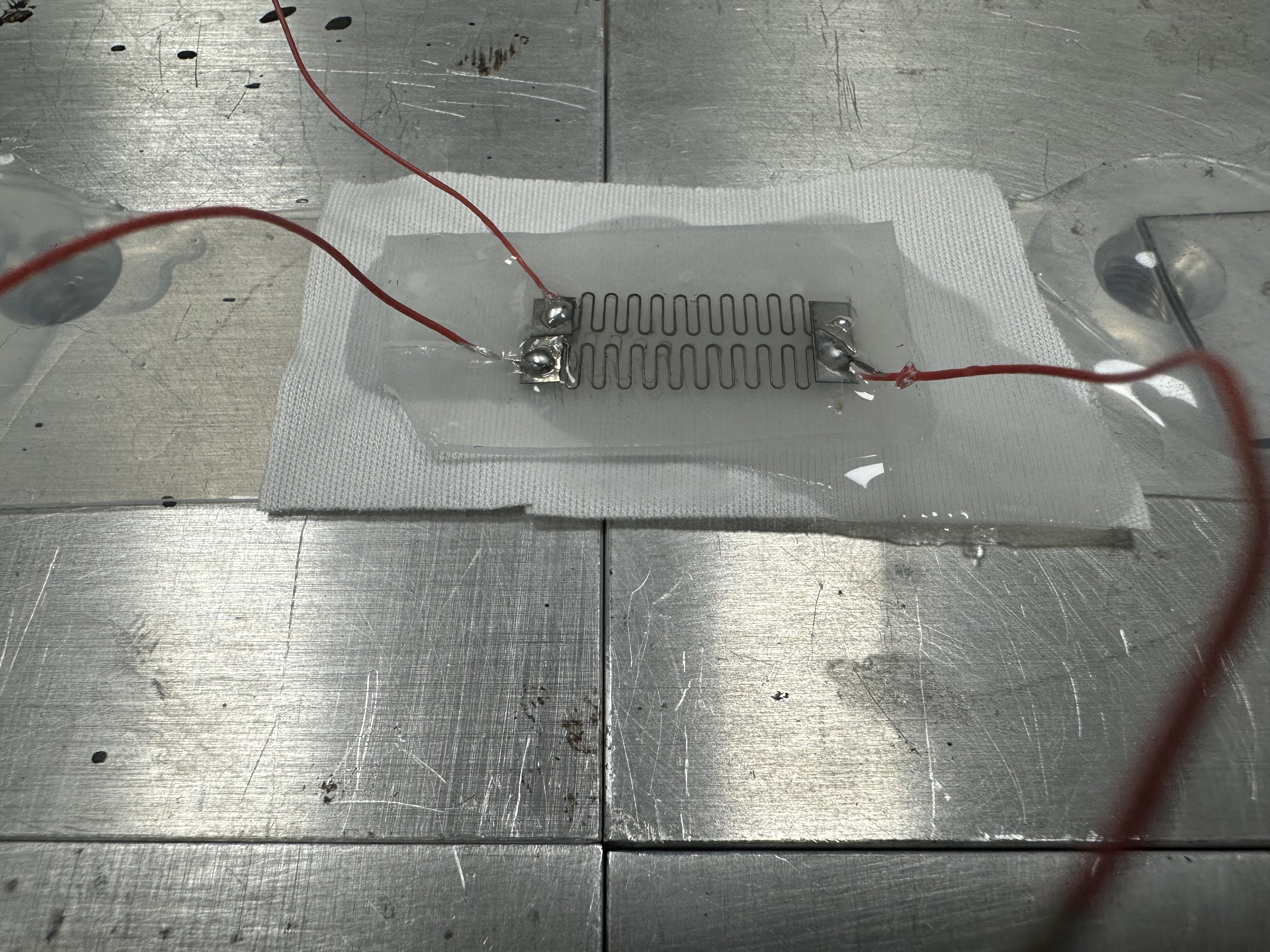

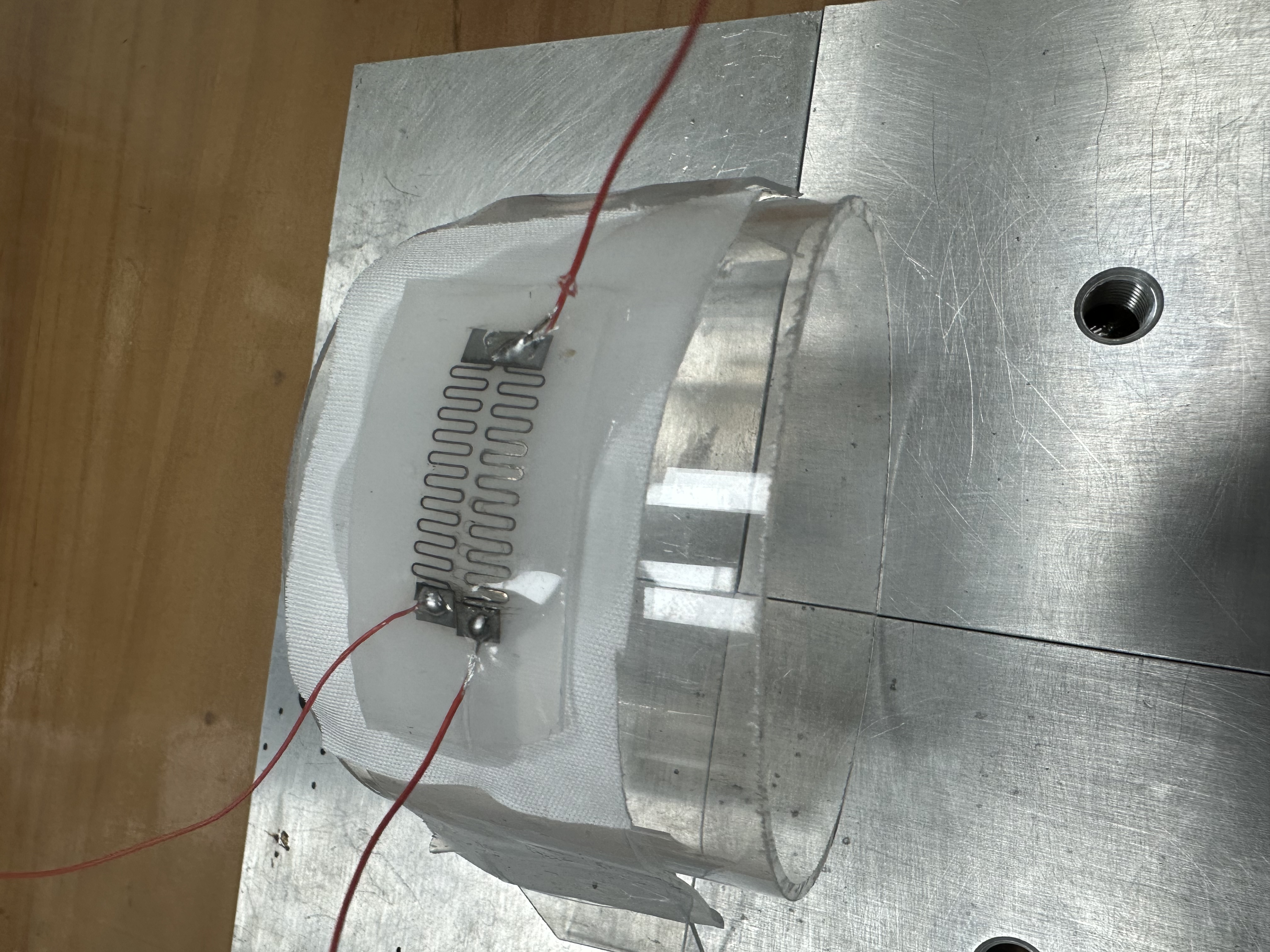


**Figure S11. Slip-simulation tests.**

**

**

**Figure S12. Determination of strain levels during gesture recognition. (a) Calibration curve obtained from tensile testing. (b) Resistance response during fist-clenching motion.**


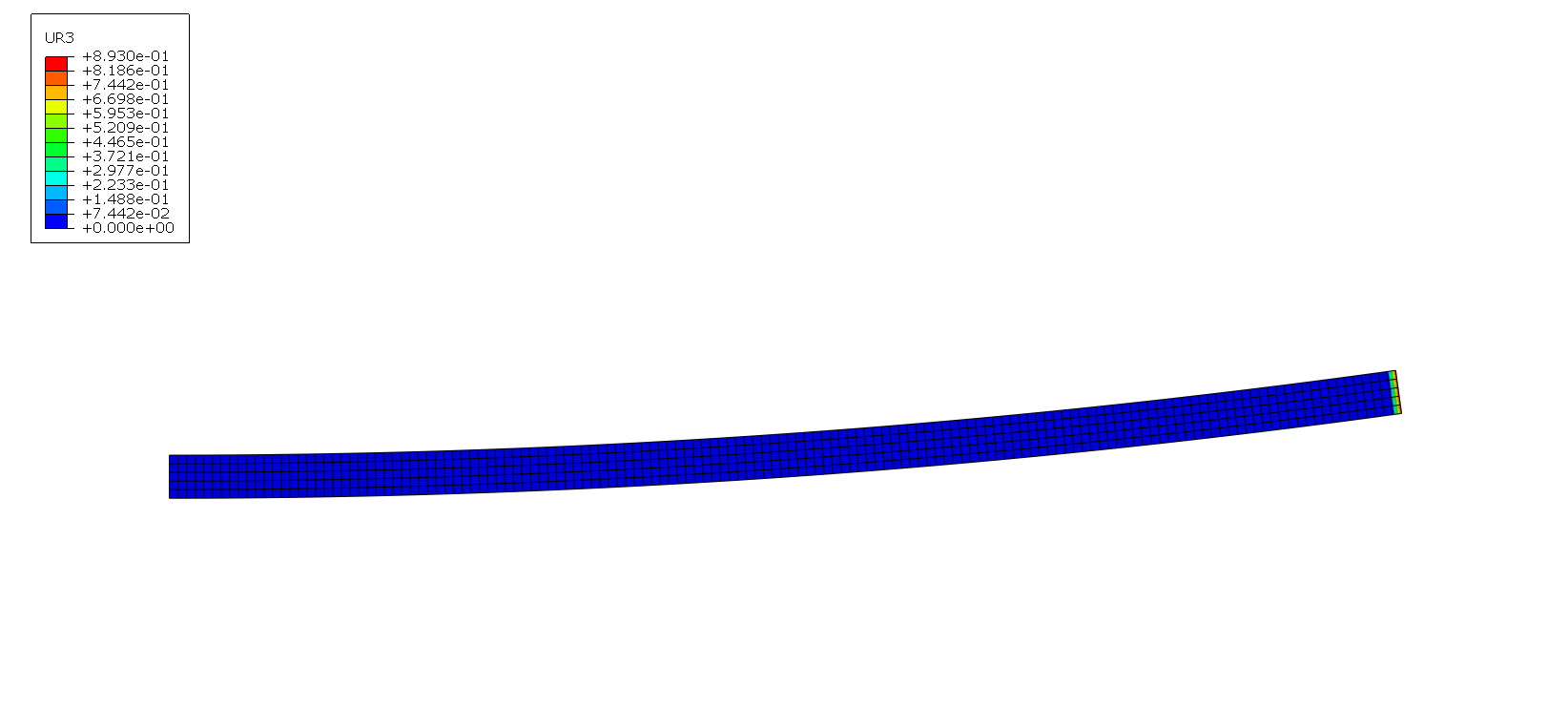


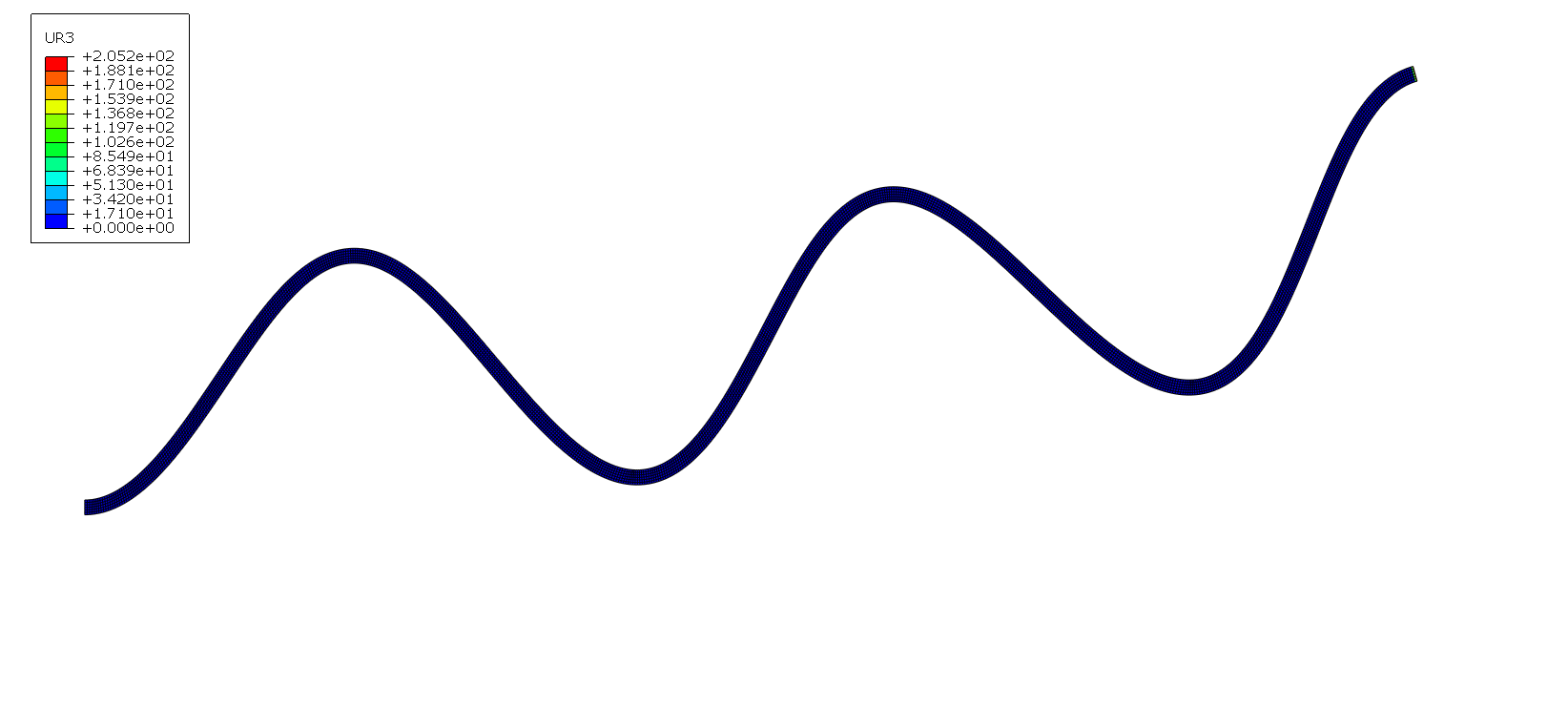


**Figure S13. Finite element analysis of bending in traditional non-stretchable curvature sensors and the developed sensors.**


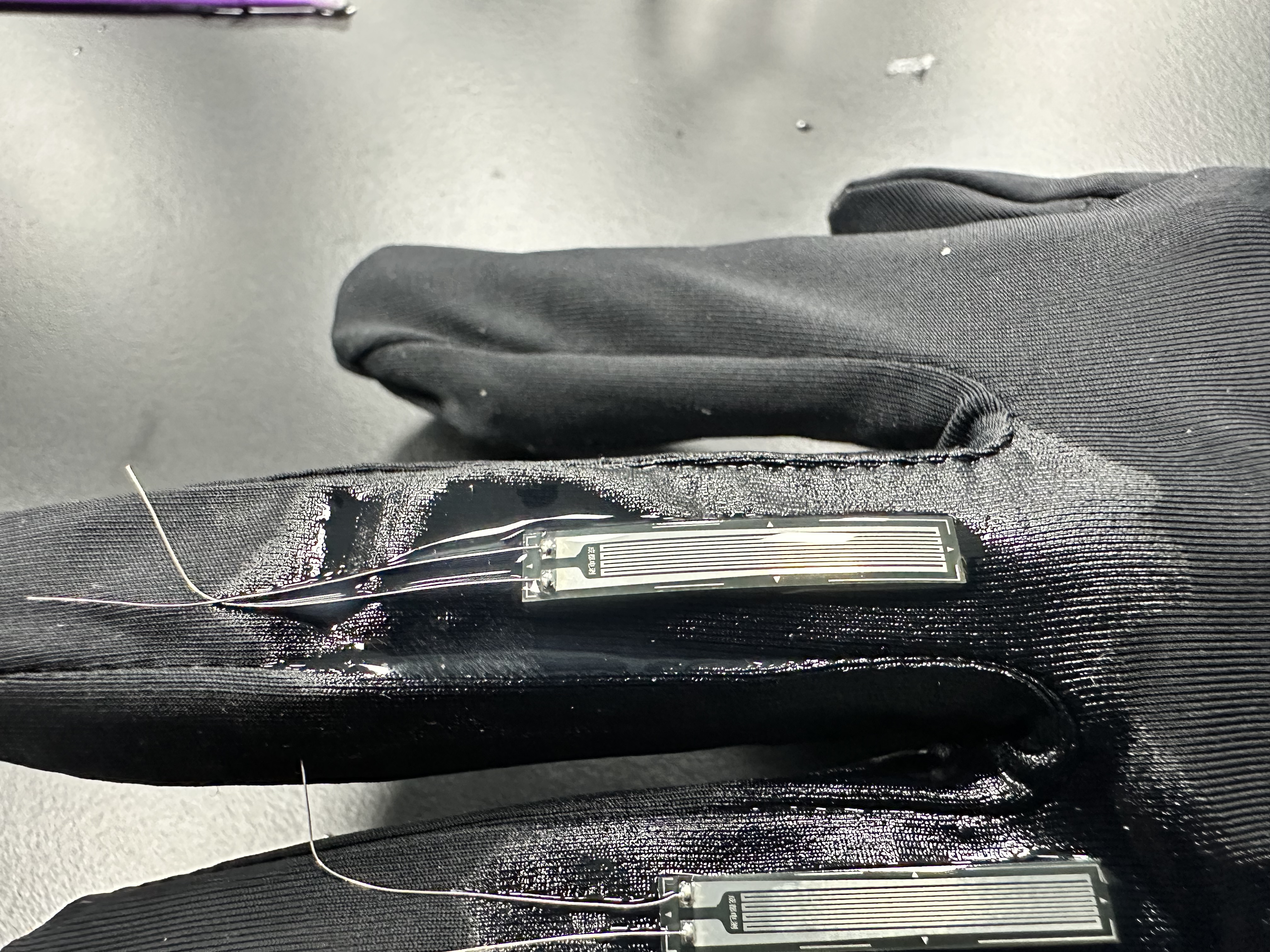

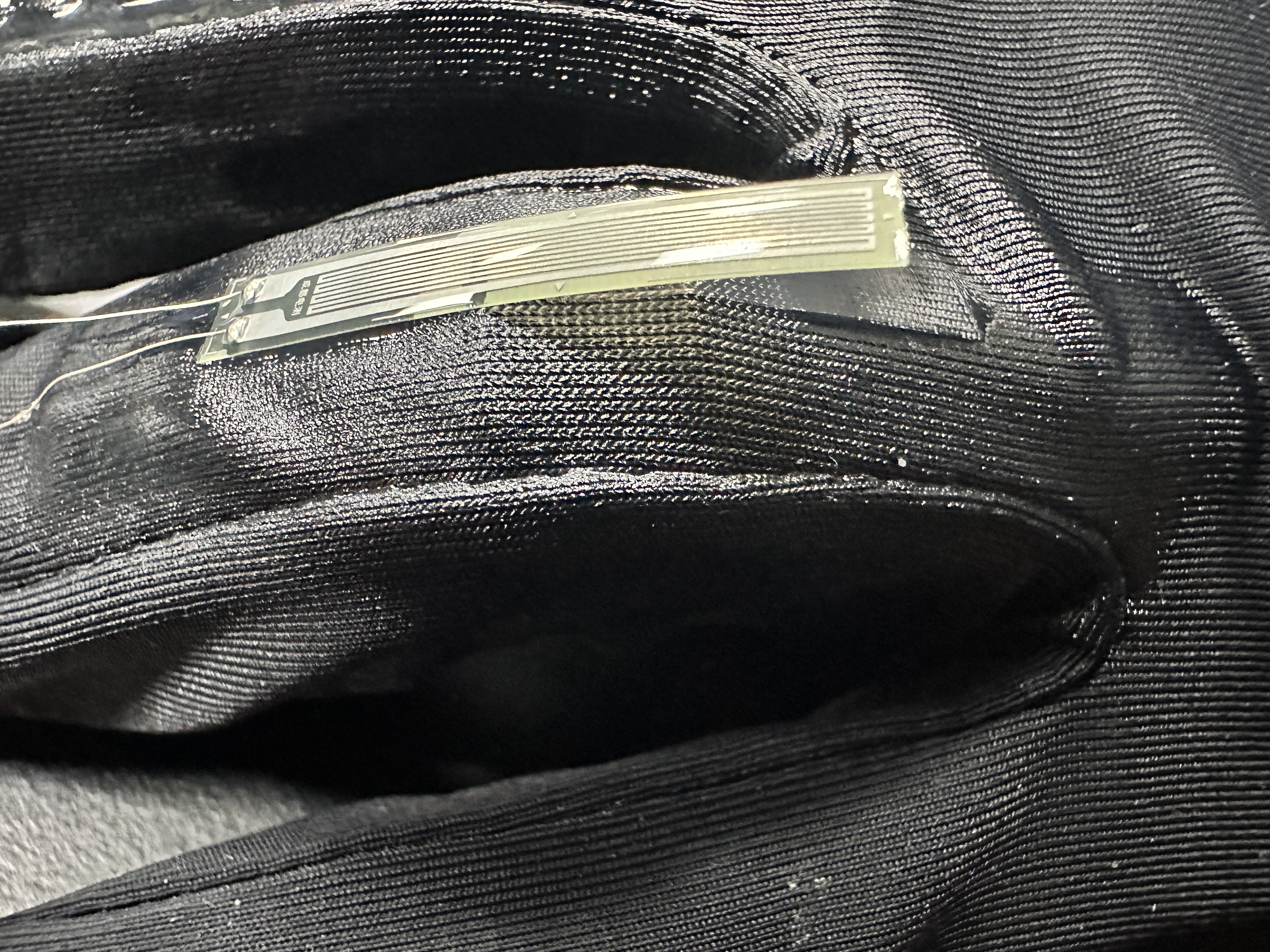


**Figure S14. During large-scale gestures, the non-stretchable sensors on the gloves exhibited wrinkling and detachment.**





**Figure S15.** **Subtle motion-distinction tests using a smart glove integrated with the proposed curvature sensors.**





**Figure S16.** **Long-term wearing and sweat-exposure stability tests under realistic usage conditions. (a) Initial signal of the unused sensor. (b) Sensor response after natural sweating induced by 45 minutes of exercise. (c) Sensor response after 12 hours of continuous daily-life wearing. (d) Sensor response after 1 hour of water immersion.**


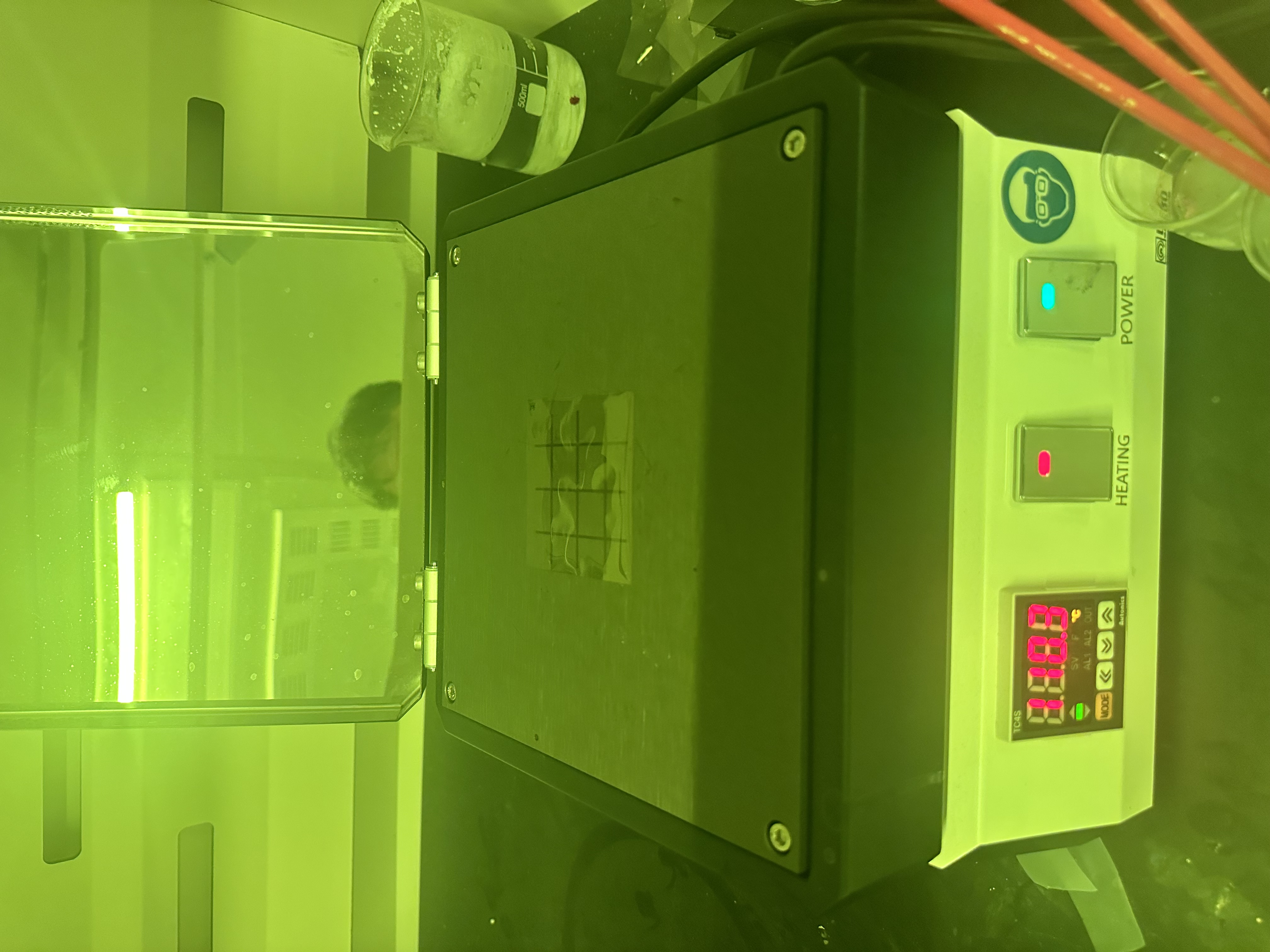


**Figure S17.** **Schematic diagram of the pre-baking of the checkered cushion film.**





**Figure S18.** **Adhesion reliability of the Kafuter flexible adhesive under realistic wearable conditions. (a) Initial adhesion state of the newly assembled device. (b) Adhesion condition after natural sweating induced by 45 minutes of exercise. (c) Adhesion condition after 12 hours of continuous daily-life wearing. (d) Adhesion condition after 1 hour of water immersion.**


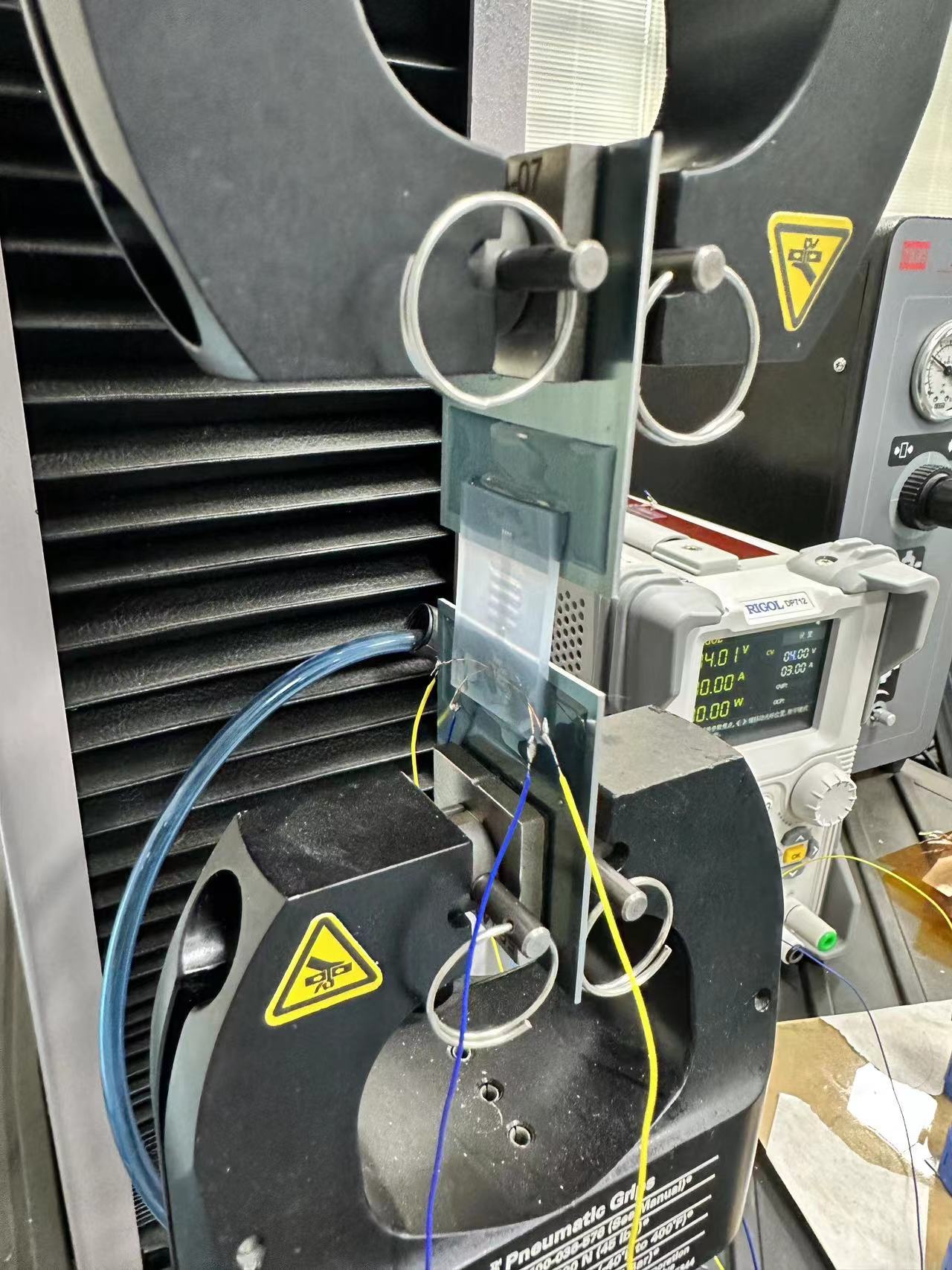


**Figure S19.** **Schematic diagram of the programmable stretch testing machine.**


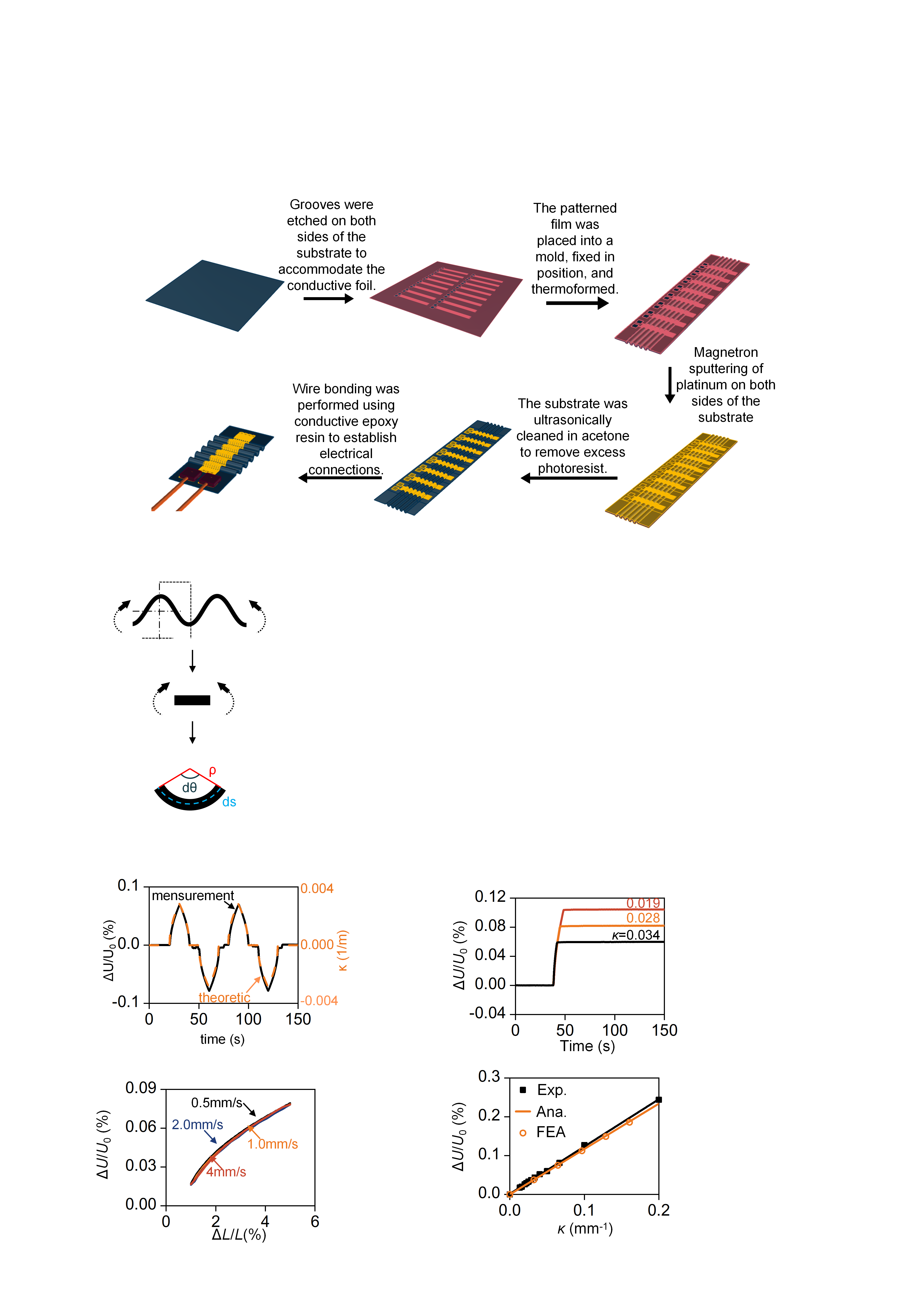


**Figure S20.** **Data diagrams of finite element analysis, experimental results, and theoretical calculations.**
